# Casposons: a new superfamily of self-synthesizing DNA transposons at the origin of prokaryotic CRISPR-Cas immunity

**DOI:** 10.1186/1741-7007-12-36

**Published:** 2014-05-19

**Authors:** Mart Krupovic, Kira S Makarova, Patrick Forterre, David Prangishvili, Eugene V Koonin

**Affiliations:** 1 Institut Pasteur, Unité Biologie Moléculaire du Gène chez les Extrêmophiles, 25 rue du Docteur Roux, 75015 Paris, France; 2 National Center for Biotechnology Information, National Library of Medicine, Bethesda, MD 20894, USA

**Keywords:** Mobile genetic elements, CRISPR-Cas system, Adaptive immunity, Transposons, Archaea, DNA polymerases

## Abstract

**Background:**

Diverse transposable elements are abundant in genomes of cellular organisms from all three domains of life. Although transposons are often regarded as junk DNA, a growing body of evidence indicates that they are behind some of the major evolutionary innovations. With the growth in the number and diversity of sequenced genomes, previously unnoticed mobile elements continue to be discovered.

**Results:**

We describe a new superfamily of archaeal and bacterial mobile elements which we denote casposons because they encode Cas1 endonuclease, a key enzyme of the CRISPR-Cas adaptive immunity systems of archaea and bacteria. The casposons share several features with self-synthesizing eukaryotic DNA transposons of the Polinton/Maverick class, including terminal inverted repeats and genes for B family DNA polymerases. However, unlike any other known mobile elements, the casposons are predicted to rely on Cas1 for integration and excision, via a mechanism similar to the integration of new spacers into CRISPR loci. We identify three distinct families of casposons that differ in their gene repertoires and evolutionary provenance of the DNA polymerases. Deep branching of the casposon-encoded endonuclease in the Cas1 phylogeny suggests that casposons played a pivotal role in the emergence of CRISPR-Cas immunity.

**Conclusions:**

The casposons are a novel superfamily of mobile elements, the first family of putative self-synthesizing transposons discovered in prokaryotes. The likely contribution of capsosons to the evolution of CRISPR-Cas parallels the involvement of the RAG1 transposase in vertebrate immunoglobulin gene rearrangement, suggesting that recruitment of endonucleases from mobile elements as ready-made tools for genome manipulation is a general route of evolution of adaptive immunity.

## Background

Cellular organisms in the three domains of life are under constant onslaught of invading mobile genetic elements (MGE), such as transposons, viruses and plasmids. Many, if not most, of these diverse selfish elements insert into the chromosomes of the cellular hosts, either as an obligate part of their life cycles or at least occasionally, and in multicellular eukaryotes constitute a substantial proportion of the host genome. For example, sequencing of the human genome has shown that transposons or relics thereof amount to 35% to 50% of the genome [1,2] and subsequent analyses have only revised these estimates upward [3,4]. Even more strikingly, in some green plants, MGE-derived DNA seems to represent more than 70% of the genome [5,6]. Although not as abundant as in eukaryotes, proviruses and other MGE constitute up to 30% of some bacterial genomes [7,8]. The effects of MGE integration vary from beneficial (gain of new phenotypic traits, such as antibiotic resistance or toxin production) to deleterious (disruption or inactivation of essential cellular genes upon MGE insertion) [7,9-11]. For most prokaryotic plasmids and viruses, the circular form of the MGE genome is inserted into specific loci (site-specific integration) of the cellular chromosome with the aid of MGE-encoded enzymes known as integrases [12]. The integrases are grouped into two major families on the basis of sequence conservation and mechanistic relatedness: (1) tyrosine recombinases use a catalytic tyrosine residue which attacks the DNA and becomes covalently linked to it during strand exchange; and (2) serine recombinases for the same purpose use a nucleophilic serine residue [12].

Transposons are DNA segments that move from one location in the host genome to another. Although several classification schemes have been proposed, based on the nature of the transposition intermediate, transposons can be generally grouped into two classes [10,13] or types [14]. Class I (or type 2) elements—also known as retrotransposons—transpose via an RNA intermediate which prior to integration is copied back to the DNA form by the element-encoded reverse transcriptase. Class II (or type 1) DNA transposons move in the genome by the so-called ‘cut-and-paste’ mechanism whereby the transposon is excised from its initial location and inserted into a new genomic locus. Most of the class II transposons possess characteristic terminal inverted repeats (TIR) but differ widely in terms of the transposases they encode, the specific mechanisms of transposition, the element size and gene content [10,13,15]. Although most transposases belong to the DDE superfamily (named after two aspartate and one glutamate residues that form the catalytic triad of these enzymes) [10,13,16], some transposons encode transposases homologous to the rolling-circle replication initiation endonucleases found in numerous viruses and plasmids [17-19], to phage integrase-like tyrosine recombinases [20,21] or to the serine integrases/invertases [22]. Furthermore, some bacterial and eukaryotic viruses encode transposases that are involved in the integration of the viral genome into the host chromosome, thereby partially blurring the distinction between different MGE types [23-25].

A distinct group of MGE consists of large (15 to 20 kb), self-synthesizing DNA transposons, called Mavericks or Polintons [26,27]. The defining feature of Polintons/Mavericks is that they encode their own protein-primed type B DNA polymerase which is most likely involved in the transposon replication (hence ‘self-synthesizing’ transposons) [26]. In addition, these transposons encode several hallmark viral proteins, the genome packaging ATPase and protease. Recently, we have shown that Polintons/Mavericks also encode major and minor capsid proteins, suggesting that these elements combine features of bona fide viruses and transposons [24]. Polintons/Mavericks are widespread in diverse unicellular and multicellular eukaryotes [26,27]. In contrast, no such self-synthesizing DNA transposons have been described in prokaryotes.

To survive the proliferation of various MGE and to maintain genetic integrity, cellular organisms have evolved numerous defense lines, including a variety of innate and adaptive immunity mechanisms [28-30]. Although once considered to be characteristic exclusively of animals, adaptive immunity has been recently discovered in bacteria and archaea [30-33]. This system consists of arrays of clustered regularly interspaced short palindromic repeats (CRISPR) and CRISPR-associated proteins (Cas) and elicits interference against foreign nucleic acids by degrading them in a sequence-specific fashion. The specificity is ensured by the unique spacers homologous to viral or plasmid DNA and integrated into the CRISPR loci. The action of the CRISPR-Cas system can be divided into three stages. The first stage, called adaptation, involves insertion of foreign DNA spacers into the CRISPR repeats. This step is mediated by the two most conserved core proteins of the CRISPR-Cas system, Cas1 and Cas2 [34-36]. Although the mechanistic details of adaptation remain poorly understood, it has been demonstrated that Cas1 is the endonuclease responsible for the excision of the protospacer from the foreign DNA and its insertion into the CRISPR cassette [36-40]. During the second stage, expression and processing, the CRISPR locus containing the arrays of spacers is transcribed, producing a long pre-crRNA (CRISPR RNA), which is subsequently processed by Cas proteins into short guide crRNAs. The final stage is called interference and involves degradation of the alien DNA or RNA by the Cas enzymatic machinery guided by the bound crRNA [30,31,35]. Phylogenomic analyses of the Cas proteins from diverse archaea and bacteria yielded a wealth of information on the diversity and evolution of the CRISPR-Cas immunity [33]. However, it remains unclear how this sophisticated defense system emerged in the first place.

Here, we describe the discovery and characterization of a new superfamily of MGE that possess several features resembling the eukaryotic self-synthesizing DNA transposons but are integrated in the genomes of various archaea and some bacteria. Along with family B DNA polymerases (PolB), that are related either to viral protein-primed PolBs or to typical archaeal PolBs, these elements, which we denote ‘Casposons’, encode Cas1 proteins of a distinct subfamily. We propose that, different from other known MGE, casposons utilize Cas1 endonucleases for integration into the host genomes via a mechanism resembling that of spacer integration by CRISPR-Cas systems. Given that Cas1 is a key enzyme of the CRISPR-Cas immunity and considering the deep branching of casposon homologs in the Cas1 phylogeny, casposons appear to have played a pivotal role in the origin of the adaptive immune system in prokaryotes.

## Results

### Genomic islands containing stand-alone *cas1* genes

A recent comparative genomic survey of *cas* genes revealed two distinct groups of *cas1* genes that are not associated with CRISPR loci and form two distinct clades in the Cas1 phylogeny (hereinafter ‘Cas1-solo’) [33]. The first Cas1-solo group was exclusively found in members of the archaeal order *Methanomicrobiales* and did not show any evidence of horizontal gene transfer (HGT) whereas the second group displayed a more patchy distribution. Most of the group 2 members were from the euryarchaeal class *Methanomicrobia*; however, several representatives were also detected in members of Thaumarchaeota as well as in the hyperthermophilic euryarchaeon *Aciduliprofundum boonei* affiliated with the order *Thermoplasmatales*[33]. We hypothesized that Cas1-solo might be ancestral to the Cas1 proteins found in CRISPR-Cas systems and set out to investigate their provenance and potential function.

Previous site-directed mutagenesis study has identified four conserved residues constituting the active site of Cas1 endonucleases (E141, H208, D218 and D221 in *Escherichia coli* Cas1); alanine substitutions at any of these positions abolished the nuclease activity of the Cas1 against all substrates tested [37]. Examination of the multiple alignment of Cas1-solo protein sequences showed that none of the group 1 members had the full complement of active site residues [see Additional file 1: Figure S1a], indicating that these proteins are unlikely to be active endonucleases (or less likely, rely on a different set of catalytic residues) and evolved under different constraints than the functional Cas1 proteins. In contrast, the four catalytic residues are strictly conserved in all Cas1-solo proteins from group 2 [see Additional file 1: Figure S1b]. Therefore, for further analysis we focused on group 2 of Cas1-solo.

**Figure 1:**
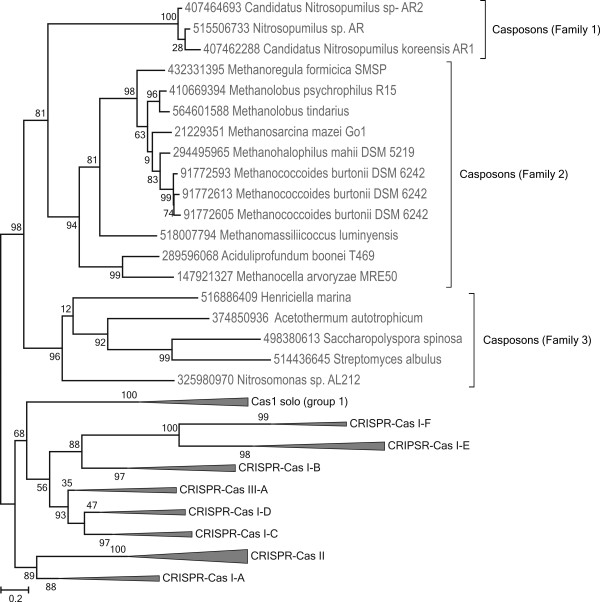
**Phylogeny of Cas1 proteins.** Cas1 proteins encoded by casposons are represented in the framework of the Cas1 sequences representing the major types of CRISPR-Cas system. All clades including Cas1 from different CRISPR-Cas systems as well as Cas1-solo group 1 were collapsed for clarity (altogether, 52 non-casposon Cas1 sequences were analyzed, a representative subset of a larger collection of Cas1 sequences analyzed previously [33]; the Cas1 sequence alignment used to generate the tree is provided in Additional file 2, whereas the tree in which all branches are expanded is shown in Additional file 1: Figure S5). Numbers at the branch points represent RELL (resampling of estimated log-likelihoods)-like local support values calculated by FastTree.

It has been noted that group 2 *cas1-*solo genes are often present in a conserved neighborhood that additionally includes genes for a PolB-like polymerase, an HNH nuclease and two helix-turn-helix (HTH) domain-containing proteins [33]. To explore the phyletic distribution of such Cas1-solo-containing genomic islands, we searched the available archaeal and bacterial genomes for co-occurrence of c*as1* and *polB* genes. This analysis identified multiple ‘genomic islands’, in addition to those previously reported [33]. In total, we detected 19 islands matching our criteria [see Additional file 1: Table S1]. In addition to archaea, such islands were detected in the genomes of several bacteria, namely *Streptomyces albulus* CCRC 11814 (*Actinobacteria*), *Henriciella marina* DSM 19595 (*Alphaproteobacteria*) and *Nitrosomonas* sp. AL212 (*Betaproteobacteria*) as well as on a genomic scaffold of an uncultured thermophilic bacterium Candidatus ‘Acetothermum autotrophicum’. Phylogenetic analysis confirmed that all newly identified Cas1 proteins belong to the same clade of Cas1-solo group 2 (Figure 1). Notably, the divergence of this Cas1 group appears to antedate the radiation of the three major types of CRISPR-Cas systems [35].

### Discovery of casposons

Gene content analysis showed that Cas1-solo-encoding genomic islands from Thaumarchaeota contain PolBs that belong to the group of protein-primed polymerases. These polymerases are encoded by various viruses and eukaryotic self-synthesizing transposons of the Polinton/Maverick family [26,27] but generally not by cellular organisms. Thus, we hypothesized that these islands represent integrated MGE, analogous to the eukaryotic self-synthesizing transposons. DNA transposons typically possess TIR and upon integration into the genome often contain a specific mark, the target site duplication (TSD) which flanks the transposon [13,15]. We investigated the Cas1 and PolB-containing genomic islands for the presence of these features and found that in nearly all cases these loci were flanked by TIRs and direct repeats which correspond to TSD [see Additional file 1: Table S1]. None of these elements contained identifiable genes for serine or tyrosine recombinases nor did they carry conserved transposase genes (see also below). The only enzyme that is consistently present in all these elements and, judged by its experimentally characterized activity, is capable of mediating the integration of the elements into the host genome is Cas1. Accordingly, we denote this new group of transposon-like elements ‘Casposons’. The conservation of *polB* genes places casposons as a new (super) family into the class of self-synthesizing large DNA transposons [14].

### TIRs, TSDs and integration sites

The unique casposon TIRs are highly variable in length (25 to 602 nucleotides, median of 56) and could be identified in all casposons, except for the three closely related elements (MetBur-C1 to C3) in the genome of *Methanococcoides burtonii* DSM 6242 [see Additional file 1: Figure S2]. Some of the TIRs contain internal palindromic sequences [see Additional file 1: Figure S2}.

The TSDs result from the fill-in repair of staggered nicks introduced by transposases at the target site upon insertion of MGE [15,41]. The length of the TSD differs depending on the transposase involved but in addition varies within as well as between transposon families [13,15]. The great majority of casposons are flanked by perfect direct repeats corresponding to TSD and ranging in length from 1 to 27 nucleotides (median of 15; Additional file 1: Table S1). In a substantial fraction of the identified casposons, one or both TIRs partially overlap with the TSDs [see Additional file 1: Table S1 and Figure S2], suggesting that, prior to integration, these casposons contained short terminal overhangs that were partially complementary to the staggered ends of the nicked target site. By contrast, the casposons in which the overlaps between the TSDs and TIRs were not present likely had blunt termini prior to integration.

Most transposons do not display strong target site preference but some are known to integrate site-specifically [42,43]. Ten casposons were found to be inserted into intergenic loci whereas for eight others, the target sites overlapped with coding sequences. The target sites of the three complete thaumarchaeal casposons were located within the 3′-distal region of the gene encoding the translation elongation factor aEF-2, whereas in five euryarchaeal casposons the target site overlapped four to seven 3′-distal nucleotides of different tRNA genes [see Additional file 1: Table S1]. Notably, eukaryotic transposons of the recently described DADA superfamily integrate site-specifically into snRNA and tRNA genes [42]. However, unlike DADA transposons, which integrate close to the anticodon loop of tRNA genes, casposons do not alter the sequence of their target genes (either tRNA or aEF-2) and are located proximal to these genes. This pattern of integration is reminiscent of the bacterial Tn7 transposon which recognizes the 3′-distal region of the highly conserved glutamine synthetase gene (*glmS*) but inserts downstream of the *glmS* coding region, preserving the integrity of the latter [43]. Such a strategy ensures that the integration of Tn7 and casposons does not disrupt genes essential for host viability, thereby ensuring successful propagation of both the host and the respective MGE.

### Casposon mobility

In most cases, when complete genome sequences are available, casposons are present in one copy per genome, consistent with their site-specific integration. However, *M. burtonii* DSM 6242 encompasses three closely related casposons (MetBur-C1 to -C3) which are adjacent on the genome [see Additional file 1: Figure S3a], suggesting recent activity of casposons in this archaeon. Notably, MetBur-C3 appears to be inactivated because two of the genes in this element contain amber mutations. Again, a parallel with the Tn7 transposon can be drawn. Tn7 is usually present in a single copy per genome. However, with lower efficiency, additional copies can be integrated into the same target site, forming islands of tandem transposons, some of which become inactivated with time [44]. *M. psychrophilus* R15 is another organism in which remnants of a second, adjacent casposon are present [see Additional file 1: Figure S3b]. The TIRs of the latter element could not be identified, suggesting that it might be in the process of deterioration. The patchy taxonomic distribution of casposons in archaeal and bacterial genomes as well as their amplification in certain organisms suggests that they are active mobile elements. However, the possibility that the amplification of casposons in *M. burtonii* DSM 6242 and *M. psychrophilus* R15 genomes is a result of segmental duplication cannot be ruled out. Experimental study of casposon integration and excision, as well as analysis of many more complete archaeal genomes, is necessary to provide definitive answers regarding the mobility of these MGE.

### Classification of casposons

**Figure 2:**
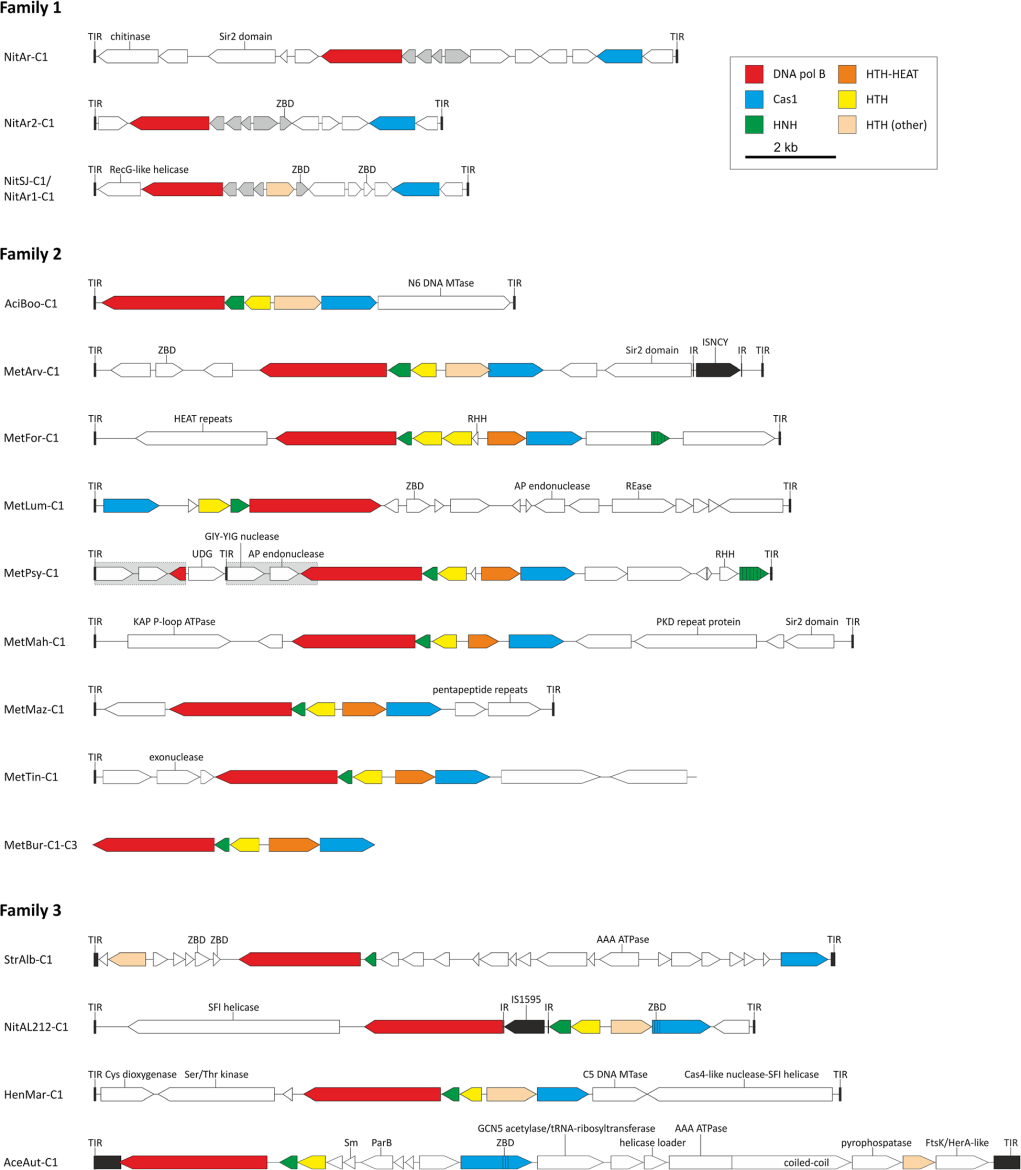
**Casposon genome maps**. The three families of casposons are indicated. The precise nucleotide coordinates of depicted casposons can be found in Additional file 1: Table S1. Predicted protein-coding genes are indicated with arrows, indicating the direction of transcription. The color key for the designation of the common genes is shown in the top right area of the figure. ‘HTH (other)’ denotes proteins that are not orthologous but nevertheless contain HTH domains. Terminal inverted repeats (TIR) are shown with black rectangles and their sequences are shown in Additional file 1: Figure S2. The grey boxes outlined with a broken line in MetPsy-C1 depict duplicated regions. The striped green arrows represent genes encoding divergent HNH proteins. Abbreviations: ZBD, zinc-binding domain-containing protein; HNH, HNH family endonuclease; HTH, helix-turn-helix proteins; MTase, methyltransferase; RHH, ribbon-helix-helix protein; REase, restriction endonuclease; AP, apurinic/apyrimidinic; UDG, uracil-DNA glycosylase; SFI, superfamily I; IR, inverted repeat.

Based on the gene content, taxonomic distribution and specific relationships between the Cas1 proteins, casposons can be classified into three families (Figure 2). All four family 1 casposons are found in the genomes of different ammonia-oxidizing species of the thaumarchaeal genus *Nitrosopumilus* isolated from marine sediments [45,46]. The NitSJ-C1 casposon from *Nitrosopumilus* sp. SJ is nearly identical to NitAR1-C1 from *Candidatus* Nitrosopumilus koreensis AR1, except for two single-nucleotide deletions in the latter. Otherwise, similarity between family 1 casposons is limited to five universally present genes, including *cas1*, *polB* and three small genes of unknown function (Figure 2). Cas1 proteins of family 1 casposons form a separate clade in the phylogenetic tree (Figure 1) and are more compact compared to the homologs from other casposons, with no additional protein domains (see below).

**Figure 3:**
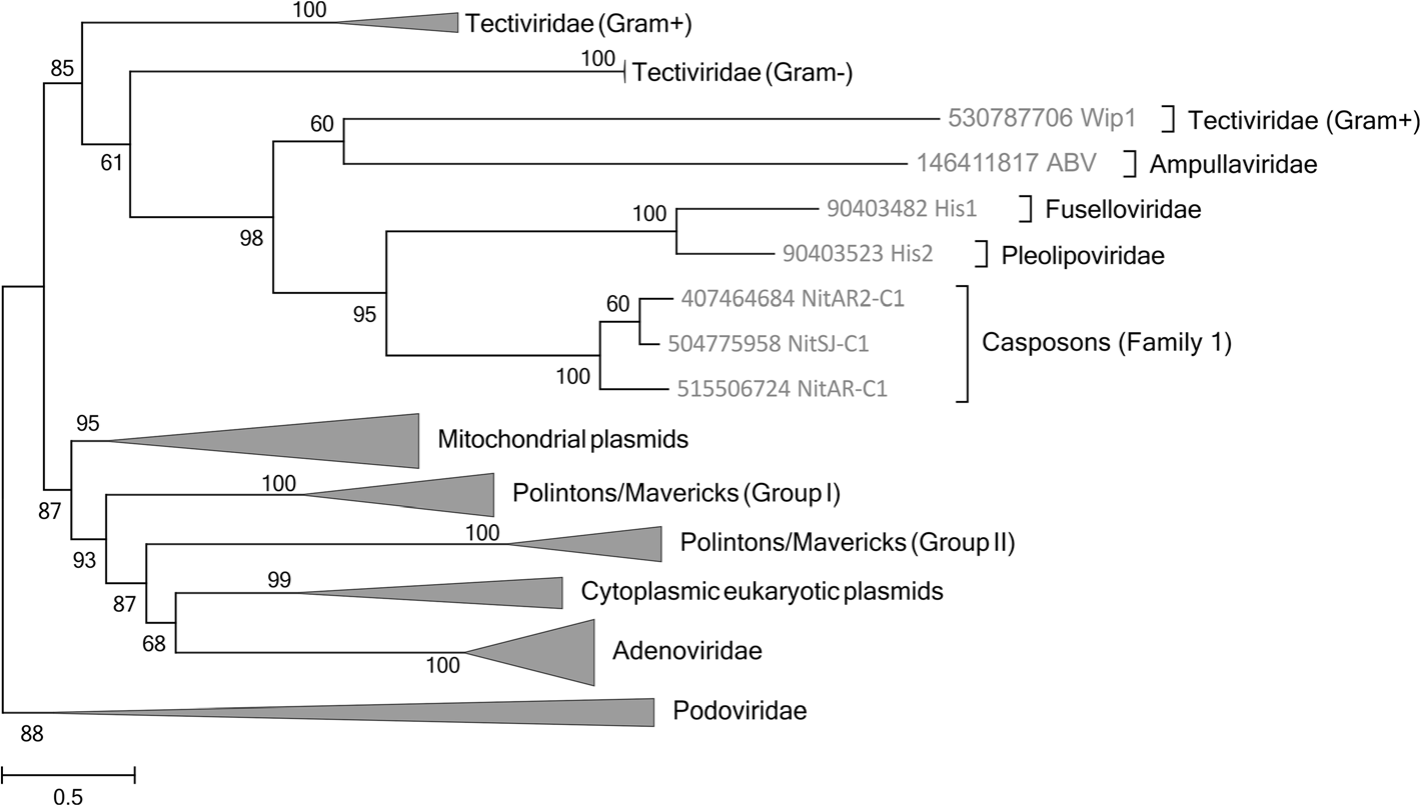
**Phylogeny of protein-primed B family DNA polymerases.** Clades that are only distantly related to the casposon-encoded proteins were collapsed. The tree is rooted with phi29-like bacteriophages of the *Podoviridae* family. Numbers at the branch points represent RELL (resampling of estimated log-likelihoods)-like local support values calculated by FastTree.

The defining feature of family 1 casposons is that they carry a gene for a protein-primed PolB. To investigate the relationship between family 1 casposons and other protein-primed PolB-encoding MGE, in particular the eukaryotic Polinton/Maverick transposons, we performed a phylogenetic analysis of the corresponding PolBs from a wide range of viruses, plasmids and transposons (Figure 3). In the resulting tree, the casposon PolBs form a sister group to PolBs from the halophilic archaeal viruses His1 and His2 [47], and this archaeal clade is embedded deep within the clade of prokaryotic MGE that in addition includes several viral families. Although initially considered to be related (mainly due to the presence of homologous *polB* genes), recent analysis has shown that His1 and His2 belong to different virus families [48,49], suggesting that the *polB* genes have been acquired independently by the ancestors of the two viruses. Although currently available data do not allow one to unequivocally infer the directionality of the gene transfer, it seems likely that family 1 casposons were the donors of the PolB gene for both His1 and His2 viruses. Clearly, more viral and casposon sequences are needed to ascertain the directionality of *polB* gene transfer between these different types of elements. More importantly, phylogenetic analysis of the PolB proteins (Figure 3) shows that despite sharing a number of features, including size, presence of TIRs and genes for protein-primed PolB, casposons are not related to the eukaryotic self-synthesizing transposons by descent but rather are analogous to them. Indeed, the only feature shared between casposons and Polintons/Mavericks is that both types of elements are predicted to replicate using self-encoded DNA polymerases which belong to the same family but do not form a clade (Figure 3). Nevertheless, this shared property defines the class of self-synthesizing large DNA transposons [13-15].

Family 2 casposons are present in diverse members of the archaeal phylum Euryarchaeota [see Additional file 1: Table S1], including the unclassified human gut-associated methanogen *Methanomassiliicoccus luminyensis* B10 [50] as well as the hyperthermoacidophile *A. boonei* T469 [51]. PolBs encoded by casposons of family 2 are related to the PolB3 family of typical archaeal RNA-primed DNA polymerases [see Additional file 1: Figure S4] [52,53]. The casposon polymerases form a sister group to a small clade of PolBs from *Thermoproteales* (phylum *Crenarchaeota*). Notably, in the latter clade the *polB* gene of *Ignisphaera aggregans* DSM 17230 is located within an integrated mobile element which is unrelated to casposons and carries a gene for a tyrosine integrase (KSM, MK, EVK, unpublished work). This observation suggests that, as with the family 1 casposons, there could have been exchange of PolB genes between family 2 casposons and other types of MGE. Cas1 proteins of all family 2 casposons contain a conserved C-terminal fusion of an HTH domain which is not found in any other Cas1 proteins. Notably, a similar HTH domain is also found in the C-termini of PolBs of family 2 casposons. Although family 2 casposons vary considerably in size (6 to 16 kb) and gene content, most of them share a core of five genes which encode Cas1, PolB, an HNH endonuclease and two distinct HTH proteins (Figure 2). One of the conserved HTH proteins contains a C-terminal HEAT repeat domain (PF02985); HEAT repeats form rod-like helical structures that mediate various protein-protein interactions [54]. The conserved HTH proteins and the HNH endonuclease might be involved in the target site recognition and subsequent casposon integration, in concert with Cas1. This mechanism of integration would resemble integration of the site-specific transposon Tn7 mentioned above. The heterotrimeric Tn7 transposase TnsABC binds the termini of the transposon whereas targeting to the specific region of the host *glmS* gene is mediated by the sequence-specific DNA-binding protein TnsD [43].

Family 3 casposons are present in the genomes of different bacteria, including an uncultivated thermophilic bacterium *Candidatus* ‘Acetothermum autotrophicum’ [see Additional file 1: Table S1]. In the Cas1 phylogeny, Family 3 casposons form a distinct clade that is a sister group to the rest of the casposons (Figure 1). By contrast, in the PolB tree, the Family 3 clade emerges from within the Family 2 casposons [see Additional file 1: Figure S4], compatible with the possibility that casposons emerged in archaea and were horizontally transferred to bacteria subsequent to the divergence of the casposon families 1 and 2. Cas1 protein of NitAL212-C1 contains a zinc-binding domain (ZBD) and a HTH domain fused to the N- and C-termini of the Cas1 domain, respectively, whereas in AceAut-C1 both ZBD and HTH are fused to the C-terminus of the Cas1 domain (Figure 2). The Cas1 proteins of StrAlb-C1 and HenMar-C1 do not contain any additional domains, similar to the Cas1 of Family 1 casposons. Three of the four group 3 casposons contain genes for the HNH endonuclease and a conserved HTH protein shared with the group 2 casposons (Figure 2). StrAlb-C1 encodes only a homolog of the HNH endonuclease although a gene for an unrelated HTH protein, which might be functionally equivalent, was also identified (Figure 2). Thus, both the PolB phylogeny and the comparison of the sets of predicted genes point to an affinity between the casposon families 2 and 3.

### Casposon gene repertoire

Casposons vary greatly in terms of gene content, both within and between the three families described above, and carry many lineage-specific genes. Virtually all of the genes for which functions could be inferred are predicted to be involved in various DNA manipulations. Three consistent themes could be discerned among the products of the casposon genes (Figure 2 and Additional file 1: Table S2).

The first group of proteins includes predicted nucleases potentially involved in casposon integration/excision. In addition to the Cas1 endonuclease, the hallmark casposon protein, this group includes the HNH endonuclease that is present in all Family 2 and Family 3 casposons and is likely to cooperate with Cas1 in the integration and excision processes. MetLum-C1 and MetPsy-C1 contain genes for apurinic/apyrimidinic (AP) endonucleases, whereas MetPsy-C1 also encodes a GIY-YIG nuclease and a uracil-DNA glycosylase (UDG) that might be involved in the repair of the termini following casposon integration. MetTin-C1 encodes an exonuclease which could contribute to the processing of the casposon termini.

The second group of casposon proteins is implicated in DNA replication. Besides the two types of PolB genes, many casposons carry genes for various helicases which might assist during the replication of the casposon DNA. Notably, AceAut-C1 encodes not only a HerA-like helicase but also a putative DnaC-like helicase loader as well as an additional protein containing the AAA + ATPase domain, which is found in many helicases, including MCM [55]. HenMar-C1 encodes a, so far, unique fusion protein containing an N-terminal nuclease domain related to the Cas4-like proteins of the CRISPR-Cas systems and a C-terminal superfamily I helicase domain.

The third category consists of various small DNA-binding proteins containing HTH, ZBD or ribbon-helix-helix (RHH) domains. Various combinations of these proteins are encoded in most casposons (Figure 2), and it cannot be ruled out that some of the uncharacterized small proteins contain highly derived versions of DNA-binding domains. These proteins could contribute to both integration/excision and replication of the casposons, and in addition, some of them might regulate expression of casposon and/or host genes.

In addition, casposons encode various enzymes some of which are associated with genome, RNA or chromatin modification whereas others are implicated in defense functions and metabolic processes [see Additional file 1: Table S2]. Three casposons encode highly divergent Sir2-like proteins; in *Sulfolobus*, Sir2 has been shown to deacetylate the major archaeal chromatin protein, Alba, thereby modulating the chromatin structure [56]. Although the casposon-encoded Sir2-like proteins might also function as deacetylases, alternative enzymatic activities of these derived Sir2 homologs cannot be ruled out. By contrast, AmiAut-C1 encodes a unique fusion protein containing an N-terminal GCN5 acetyltransferase domain and a C-terminal queuine tRNA-ribosyltransferase domain. In addition, AciBoo-C1 and HenMar-C1 encode N6 and C5 DNA methyltransferases, respectively, whereas MetLum-C1 encodes a putative restriction endonuclease. Other notable proteins encoded by casposons include a KAP family P-loop ATPase [57] (MetMah-C1), Ser/Thr kinase (HenMar-C1) and a Sm-like RNA-binding protein (AmiAut-C1).

Two casposons, MetArv-C1 and NitAL212-C1, carry insertion sequence (IS) elements of the families ISNCY and IS1595, respectively [58]. In both cases, the IS transposase genes are flanked by typical short inverted repeats and TSDs, indicating that the IS elements parasitize casposons rather than participate in their propagation. The sporadic conservation of functionally diverse genes in distinct casposons, even those that belong to the same family, indicates that, similar to viruses, casposons can horizontally acquire genes from various sources.

## Discussion

Transposons as a type of MGE are polyphyletic with respect to the enzymes mediating their transposition [10,13,15]. Here, we described a new type of mobile elements, the casposons, which appear to rely on Cas1-like endonucleases for genome integration. Structures of several Cas1 proteins have been solved [37,39,40] showing that Cas1 proteins adopt a novel structural fold, unrelated to the folds of any of the transposases described to date. Nevertheless, casposons share a number of features with known DNA transposons. On the one hand, they appear to behave as site-specific transposons, akin to the bacterial transposon Tn7 [43,44]. On the other hand, the molecular structure of casposons is highly reminiscent of the eukaryotic self-synthesizing DNA transposons of the Polinton/Maverick superfamily [26,27]. Similar to the Polintons/Mavericks, casposons possess TIRs and encode their own DNA polymerase genes which in family 1 casposons belong to the same, protein-primed class as the polymerase of Polintons/Mavericks.

**Figure 4:**
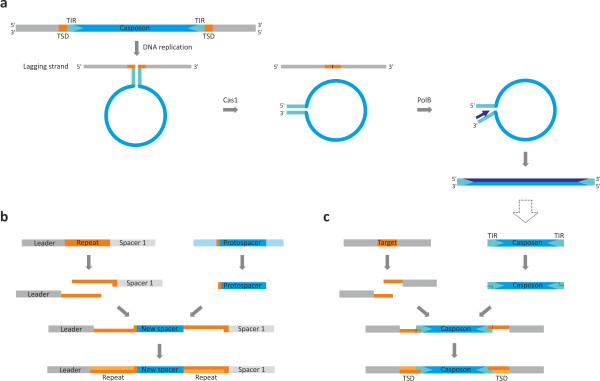
**Proposed mechanisms of casposon replication and integration. (a)** Mechanism of casposon DNA excision and replication. **(b)** Mechanism of spacer acquisition by CRISPR-Cas system. Adapted from [34]. **(c)** Proposed mechanism of Cas1-mediated casposon integration. See text for details. Subterminal regions within TIRs outlined with the broken line indicate that processing occurs only in a fraction of casposons. See text for details. Abbreviations: TIR, terminal inverted repeats; TSD, target site duplication.

Based on the model previously proposed for Polinton/Maverick transposons [26], we hypothesize that casposon DNA replication proceeds via a single-stranded (ss) DNA intermediate and primarily depends on the casposon-encoded PolB (Figure 4a). First, during cellular DNA replication, the casposon sequence is likely to loop-out on the lagging strand due to the formation of a double-stranded stem involving the TIR sequences. The next stage involves Cas1-catalyzed excision of the casposon. Importantly, Cas1 from *E. coli* has been shown to act efficiently on different branched DNA substrates [37], including the splayed-arm duplex DNA which is similar to the looped-out casposon intermediate depicted in Figure 4a. The TIRs of the excised ssDNA casposon would form the panhandle structures which serve as the replication origin in various viruses and plasmids encoding protein-primed PolBs [59,60]. In the latter systems, replication is primed by a MGE-encoded protein that covalently binds to the 5′-terminus of the nascent strand. This is likely to also be the case for the group 1 casposons which encode protein-primed PolBs (Figure 3). Terminal proteins are known to be highly divergent but are typically encoded immediately upstream of the *polB* genes or as N-terminal fusions of the PolB proteins [59-61]. All family 1 casposons share an appropriately positioned conserved gene which could encode a terminal protein (Figure 2). Family 2 and 3 casposons encode PolBs that are more closely related to the typical archaeal RNA-primed PolBs, suggesting that their replication is primed by the host primase. Eventually, a new double-stranded casposon copy is synthesized through the concerted activities of PolB and accessory host- and/or casposon-encoded replication proteins, such as helicases (Figure 4a).

Given the presence of a PolB gene in all casposons, we propose to classify these elements as the second superfamily within the class of self-synthesizing DNA transposons, in addition to the Polintons/Mavericks [14]. Most of the polintons show a distinct virus-like character, with two conserved genes encoding major and minor capsid proteins, suggesting that these elements form virus particles under some circumstances and prompting their proposed re-classification as polintoviruses [24]. The casposons, however, do not encode any detectable homologs of capsid proteins and accordingly are likely to adhere to the transposon lifestyle. We define casposons as self-synthesizing MGE which rely on Cas1 for integration. Under this definition, casposons may encode distinct DNA polymerases (as is indeed the case for family 1 compared to families 2 and 3) but should possess the ability to self-synthesize.

The Cas1 endonuclease is the key player in the adaptation step of the CRISPR-Cas immunity. Consistent with its importance, Cas1 is the most stable and conserved component of functional CRISPR-Cas systems and is considered a signature gene for these defense systems [32,33,35]. A model of spacer acquisition, which integrates the available experimental data, has been proposed [30,34-36]. This model helps to predict the specific path of the Cas1-mediated integration of casposons which is also consistent with the detailed analysis of the terminal sequences of integrated casposons [see Additional file 1: Figure S2]. Figures 4b and 4c depict the parallel flows of events underlying the insertion of new CRISPR spacers and casposons, respectively. In both cases, staggered nicks are introduced into the target sequence which in the case of CRISPR-Cas corresponds to the first repeat proximal to the leader sequence. In the case of some casposons, the TIR-containing termini are processed to produce short overhangs complementary to the tips of the nicked target site (see above). In the next step, the ends of the protospacer/casposon are joined to those of the nicked target site. The observation that in CRISPR-Cas systems Cas1 is the only protein whose enzymatic activity is essential for integration of new spacers [62], a process that involves cutting and rejoining of the cellular DNA within the CRISPR repeat arrays, suggests that the casposon Cas1 also possesses both DNA cutting and joining activities. However, the latter activity of Cas1 remains to be demonstrated experimentally. Finally, the target site is fill-in repaired, completing the casposon/spacer insertion and resulting in the TSD (for casposons) or repeat duplication (in CRISPR-Cas) (Figure 4b, c).

The discovery of casposons has important evolutionary implications. The deep branching of casposon Cas1 homologs within the global Cas1 phylogeny (Figure 1) is compatible with the possibility that the Cas1 family of endonucleases emerged in the context of mobile elements and only later was adapted for cellular defense. Consequently, we propose that casposons played a pivotal role in the origin of prokaryotic CRISPR-Cas immunity. The origin of Cas1 appears not to be the only contribution of transposable elements to the evolution of CRISPR-Cas. Indeed, recent comparative genomic analysis of the type II CRISPR-Cas systems has shown that Cas9, the key protein of these systems involved in the RNA processing and interference stages, most likely, also evolved from a distinct class of transposon proteins [63].

It has been previously hypothesized that the CRISPR-Cas system originated in archaea [32], and the present observations on the likely archaeal origin of casposons appear compatible with this hypothesis. However, it cannot be ruled out that similar to some other MGE, casposons are even more ancient and antedate advanced cellular life forms [23].

Strikingly, transposons can also be placed at the root of adaptive immunity in eukaryotes. The RAG1 protein, which plays a central role during the V(D)J recombination, was derived from the DDE transposase of *Transib* transposons [64]. The parallel contribution of transposons to the origin of adaptive immunity in prokaryotes and eukaryotes emphasizes that MGE are the molecular architects behind some of the major evolutionary innovations of their hosts, in particular, the cellular defense systems [23,65]. More specifically, given the mechanistic similarity between MGE transposition and integration, on the one hand, and insertion of spacers by the CRISPR-Cas system and immunoglobulin gene rearrangement, on the other hand, integrases and transposases appear to be ready-made tools that can be recruited and utilized by adaptive immunity systems.

## Conclusions

The diversity of MGE is astounding and is far from being fully explored. This state of affairs is well illustrated by the discovery of casposons described here. Casposons constitute the second superfamily of self-synthesizing transposon-like MGE, beside the eukaryotic Polinton/Maverick transposons, and are the first representatives of this class of elements in prokaryotes. Different MGE have evolved a number of unrelated molecular mechanisms to perform similar tasks that ensure their propagation within the host cells. The casposons, so far, are unique as the only group of MGE that apparently rely on Cas1 endonucleases, a key component of the prokaryotic CRISPR-Cas defense system, for insertion into and excision from the host genome. The perennial arms race between cellular organisms and various MGE seems to be one of the major driving forces underlying the evolution of both interacting parties and it is becoming increasingly clear that cells and MGE exchange molecular inventions that emerge in the process of this evolutionary struggle. The adaptive immunity of both prokaryotes and eukaryotes apparently evolved via recruitment of recombinases from distinct MGE, the casposons and the *Transib* family transposons, respectively. Although this route of evolution seems paradoxical given that MGE are the primary targets of the immunity systems, it is becoming clear that throughout the course of evolution, MGE have served as a rich source of naturally evolved tools for cellular genome engineering that had a major impact on the diversification of cellular organisms.

## Methods

Casposon protein sequences were analyzed using PSI-BLAST [66], CD-Search [67], and HHpred [68]. Inverted and direct repeats flanking the casposons were analyzed using Unipro UGENE [69]. The palindromic repeats within the casposon TIR sequences were identified using Mfold [70]. Insertion sequences were analyzed using ISfinfer [71]. Multiple sequences alignments were built using Promals3D [72] and Muscle [73]. The Polinton/Maverick PolB sequences were recovered from the Repbase Update database [74]. For phylogenetic analysis, gapped columns (more than 30% of gaps) and columns with low information content were removed from the alignment [75]. Phylogenetic analysis was carried out by using FastTree [76], with the Jones–Taylor–Thornton model of amino acid evolution and γ-CAT estimation of evolutionary rates across sites. The trees were visualized using MEGA6 [77]. For the Cas1 phylogeny, Cas1 protein sequences representing all major types and subtypes of the CRISPR-Cas systems were obtained from [33] and supplemented with the casposon-encoded Cas1 protein sequences. The Cas1 sequence alignment used to generate the tree is provided in Additional file 2.

## Competing interests

The authors declare they have no competing interests.

## Authors’ contributions

MK, KSM, PF, DP and EVK analyzed the data; MK and EVK wrote the manuscript. All authors read and approved the final manuscript.

## Supplementary Material

Additional file 1 **The file contains Figures S1 to S5 and Tables S1 and S2. Figure S1.** Multiple sequence alignments of the two groups of Cas1-solo proteins. **Figure S2.** Analysis of the casposon terminal inverted repeats and target site duplications. **Figure S3.** Genomic loci showing the amplification of casposons. **Figure S4.** Phylogeny of RNA-primed type B DNA polymerases. **Figure S5.** Phylogeny of Cas1 proteins. **Table S1.** Major characteristics of bacterial and archaeal casposons. **Table S2.** Annotation of the casposons. Click here for file

Additional file 2 **The file contains the multiple sequence alignment of Cas1 proteins used to generate the phylogenetic trees shown in Figure **1** and Figure S5.** Click here for file
