# Supplementary material for: Casposons: a new superfamily of self-synthesizing DNA transposons at the origin of prokaryotic CRISPR-Cas immunity
Source: BMC Biol. 2014 May 19;12:36. doi: 10.1186/1741-7007-12-36 (PMC4046053; doi:10.1186/1741-7007-12-36)
Supplement: Additional file 1 — The file contains Figures S1 to S5 and Tables S1 and S2. Figure S1. Multiple sequence alignments of the two groups of Cas1-solo proteins. Figure S2. Analysis of the casposon terminal inverted repeats and target site duplications. Figure S3. Genomic loci showing the amplification of casposons. Figure S4. Phylogeny of RNA-primed type B DNA polymerases. Figure S5. Phylogeny of Cas1 proteins. Table S1. Major characteristics of bacterial and archaeal casposons. Table S2. Annotation of the casposons. [file 1741-7007-12-36-S1.pdf]

SUPPLEMENTARY FIGURES

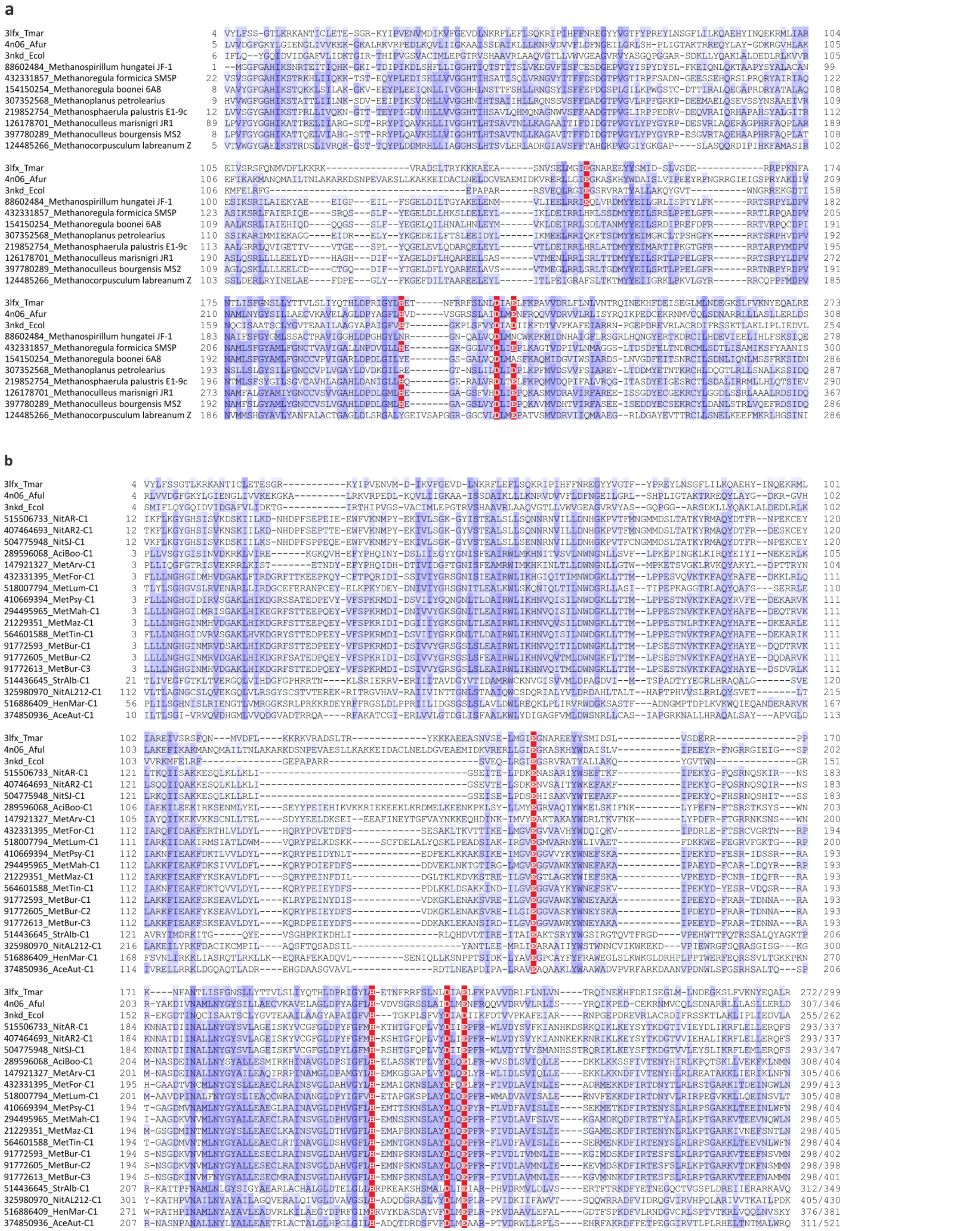

Figure S1. Two groups of Cas1-solo proteins. Multiple sequence alignment of Cas1-solo proteins belonging to groups 1 (a) and 2 (b) with three CRISPR/Cas-associated homologs for which X-ray structures are available. Positions corresponding to the four active site residues are highlighted with red background. The alignment is colored according to sequence conservation (Blosum62 matrix). The sequences are indicated by their GenBank or PDB identifiers. Tmar, *Thermotoga maritime*; Aful, *Archaeoglobus fulgidus*; Ecol, *Escherichia coli*.

NitAR-C1_Nitrosopumilus sp. AR (NZ_AJVJ01000035)

```
TIR-L (incomplete)GCCCCCTTTCTAGG
TIR-R (incomplete)GCCCCCTTTCTAGG
```

NitAR1-C1_Candidatus Nitrosopumilus koreensis AR1 (NC_018655)

```
DR   TTTATTGATAAAGAGTAAAAA
TIR-L             AGTAAAAATAGAATCCCCCCATTCCGTTCCTAATTCACTGCTGTTTTTCGTTATGACTAGAGAATGAT
TIR-R             AATAAAAATAGAATCCCCCCATTCCGTTCCTAATTCACTGCTGTTTTTCGTTATGACTAGAGAATGAT
```

NitAR2-C1_Candidatus_Nitrosopumilus_AR2_(NC_018656)

```
DR               TT
TIR-L             TCCCCCCATTTCGTGCTTAACTCACTGCTGTTTTTCGTTATGACTAGATAATTACGTGAATTATGATTTTT
TIR-R             TCCCCCCATTTCGTGCTTAACTCACTGCTGTTTTTCGTTATGACTAGATAATTACGTGAATTATGATTTGT
                                                                 ----->     <-----
```

NitSJ-C1_Nitrosopumilus sp. SJ (NZ_AJVI01000008)

```
DR   TTTATTGATAAAGAGTAAAAA
TIR-L             AGTAAAAATAGAATCCCCCCATTCCGTTCCTAATTCACTGCTGTTTTTCGTTATGACTAGAGAATGAT
TIR-R             AATAAAAATAGAATCCCCCCATTCCGTTCCTAATTCACTGCTGTTTTTCGTTATGACTAGAGAATGAT
```

AciBoo-C1_Aciduliprofundum boonei T469 (NC_013926)

```
DR     CCCCACTACGAGGAG
TIR-L             GGAGGGGGATATATATACATCCCCTCTTAAGTTCCCTTTT
TIR-R             GGGGGGGGATATATATATATCCCCTCTTAAGTTCCCTTTT
                    ----->        <-----
```

MetLum-C1_Methanomassiliicoccus luminyensis B10 (NZ_CAJE01000015)

```
DR   CGCACTTTTGTGCGGGG
TIR-L             GGGGTAGTAGTTATCGT
TIR-R             GGGGTAGTAGTTATCAT
```

MetFor-C1_Methanoregula formicicum SMSP (NC_019943)

```
DR  AAGAAACGTATGCG
TIR-L             GGGTAATAGTTATCCCGAAAAAAATGCCACTCTTGTAAAACTGGTTCACTCAACAGCGAAGGCTCCGCAAGTGCGGCGCGCTTTTTCAAAAA
TIR-R             GGGTAATAGTTATCCCGAAAAAAATGCCACTCTTGTAAAACTGGTTCACTCAACAGCGAAGGCTCCGCAAGTGCGGCGCGCTCTTTTTAAAA
                                                                           ----- --->    <--- -- ---
```

MetArv-C1_Methanocella arvoryzae MRE50 (NC_009464)

```
DR  CCCGCTGGCTTTTA
TIR-L             GGGGATAATGATATCCCCCCATTTTTTGCCGTTTTTTCTGGACGCTCCAAACTTATTATCGCCT-AACAGAGGCTTGGTCTACGAATATTAATATGACAATAATTTTATAAAATATGATTTTGGACTTAATTTCA
TIR-R             GGGGATAATGATATCCCCCCATTTTTTGCCGTTTTTTCTTGCCATACCAAACATATTATCGCTTAA-CATCGGCTTGGTCTTTAATTATTAATATAGCAGTCTTTTTATAAAAAGTGTTTTTAGACTTAATTTCA
                  ------>    <------
```

MetPsy-C1_Methanolobus psychrophilus R15 (NC_018876)

```
TIR-L             TCATGACAATAGGAAGAACAATTTCAATTTATCTACCAGATGCCAACCCGCAAGGA
TIR-R1              ATGACAATAGGAAGAACAATTTCAATTTACCTACCAGATGCCAACCCACAGGGA
TIR-R2            TCATGACAATAGGAAGAACAATTTCAATTTACCTACCAGATGCCAACCCACAGGGA
                             -- ---  -->    <--  --- --
```

MetMah-C1_Methanohalophilus mahii DSM 5219 (NC_014002)

```
DR  CGCACCAACCAATCGGGTTATAGGTAG
TIR-R             GGGTTATAGGTAGAATTGAAAAATGCAACC
TIR-L             GGGTTATAGGTAGGATAGAAAAATACAACC
```

MetMaz-C1_Methanosarcina mazei Go1 (NC_003901)

```
DR  ATAATCTTAATGCG
TIR-L             GGGATATAGGTAACTCAAAAAAACGCAACGC
TIR-R             GGGATATAGGTAACTCAAAAAAACGCAACGC
```

MetTin-C1_Methanolobus tindarius DSM 2278 (NZ_AZAJ01000001)

```
DR  CGCATATTTTTATGGGGATATA
TIR-L                  ATAGGTAACATCCAAAAATACCACT
TIR-R                  ATAGGTAACATCTAAAAATACCACT
```

StrAlb-C1_Streptomyces albulus CCRC 11814 (NZ_AROY02000134)

```
DR   CCCCCTCCCGTATGAGGG
TIR-L             GAGGGGGAGTAGAGAGAGACCCAGATTCTTGTCCGATCGTTGCGTCTGTCAACCACTCTGACGCTCCGGGGCATGCGAACGGCCACCCTCCGTGATGACGGTGGGTGGCCGTTGGG
TIR-R             GAGGGGGAGTAGAGAGAGACCCAGATTCTTGTCCGATCGTTGCGGCTGTCAACCACTCTGGCGTTGCGGGGCATGCGAACGGCCACCCTCCGTGGTGACGGTGGGTGGCCGTTGGG
                                                                                             ----------- --->    <--- -----------
```

HenMar-C1_Henriciella marina DSM 19595 (NZ_AQXT01000002)

```
DR               T
TIR-L             CCCCTCTCCCGGATGCGGGAGGGGATAAGGGAAAGCA
TIR-R             CCCCTCTCCCGCATCCGGGAGGGGATAAGGGAAAGCA
                    -- ----->    <----- --
```

NitAL212-C1_Nitrosomonas sp. AL212 (NC_015222)

```
DR  AGCCGGTACTGCCC
TIR-L             GGGGTATGTTAACTTCTCCCAAAACGCCGTACACTT
TIR-R             GGGGTATGTTAACTTCTCCCAAAACGCCGTACACTT
```

AceAut-C1_ Candidatus 'Acetothermum autotrophicum', fosmid JFF011 C09 (AP011671)

```
DR CGTCAATTCTGGCAG
TIR-L             GGGATAGTAGTGAGGCCCTCAAAATGCGGGTCACGCCCGCTTGCTGCCCACGCTCTCCATGCAACTCTCGTTTCATGCCCGTCCCAGCATCACCGCTTGCCGCTCTCGGCTAATAACAGCA 121
TIR-R             GGGATAGTAGTGAGGCCCTCAAAATGCGGGTCACGCCCGCTTGCTGCCCACGCTCTCCATGCAACTCTCGTTTCATGCCCGTCCCAGCATCACCGCTTGCCGCTCTCGGCTAATAACAGCA 121

TIR-L             ATTCACTTTCCCCCTTCACTGCTTCTCCACTGCCGCAACCCGCGCCCCCCCCGCTCCCCTGCAATCCCCTTGCTGTCCGTTACCGTCTCCCACAGTCCGCATGCGCCACTCATCAGCAGGC 242
TIR-R             ATTCACTTTCCCCCTTCACTGCTTCTCCACTGCCGCAACCCGCGCCCCCCCCGCTCCCCTGCAATCCCCTTGCTGTCCGTTACCGTCTCCCACAGTCCGCATGCGCCACTCATCAGCAGGC 242

TIR-L             CGTTTTGTCGCTTGCTGCCCAAGGTACGTCTGTTGCGGTTTGCTGTCGTGGCGGCGGCGCTGTACAGCGCGTGCGGTGCTTGTGCGCTGCTCCTTCACTACGTTCAGGACAGGCTTCACTG 363
TIR-R             CGTTTTGTCGCTTGCTGCCCAAGGTACGTCTGTTGCGGTTTGCTGTCGTGGCGGCGGCGCTGTACAGCGCGTGCGGTGCTTGTGCGCTGCTCCTTCACTACGTTCAGGACAGGCTTCACTG 363

TIR-L             CCCCTTCCCCTTCACCTTCACTATTCCTTCGCTGCTCTTTCGCTGACGCTCAGGACATGGTTCAGGGTCAGGGCTTTGGTTCAGGACAGGCTGTTGTGGCTGCCCTGCCGTTCCTATTCTC 484
TIR-R             CCCCTTCCCCTTCACCTTCACTATTCCTTCGCTGCTCTTTCGCTGACGCTCAGGACATGGTTCAGGGTCAGGGCTTTGGTTCAGGACAGGCTGTTGTGGCTGCCCTGCCGTTCCTATTCTC 484

TIR-L             CAGCGTGCTCATGTTCTGTTTACCCTTTCGTCAGCCCTTCGCTCTGTCCGTTTCGGCCCTCTGAACAGAATCGGCTGACCAGGTTGTCCAGCCCTTCCCAGTCCAGGAGTTCCCGATC    602
TIR-R             CAGCGTGCTCATGTTCTGTTTACCCTTTCGTCAGCCCTTCGCTCTGTCCGTTTCGGCCCTCTGAACAGAATCGGCTGACCAGGTTGTCCAGCCCTTCCCAGTCCAGGAGTTCCCGGTC    602
```

Figure S2. Analysis of the casposon terminal inverted repeats and target site duplications. The figure shows the direct repeats (DR) flanking the casposons as well as both the left and right terminal inverted repeats (TIR). One of the TIRs in each pair has been reverse complemented allowing their alignment. Identical nucleotides in TIRs and the overlaps between the TIRs and DRs are shown on black background.

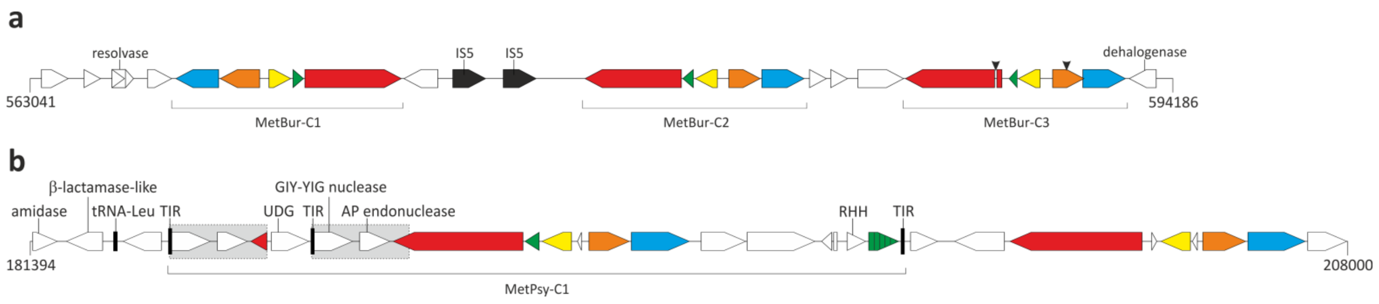

Figure S3. Genomic loci showing the amplification of casposons. (a) Locus containing the three casposons (MetBur-C1 to –C3) of *Methanococcoides burtonii* DSM 6242. Arrowheads indicate the locations of amber mutations in the genes of MetBur-C3. (b) Locus of *Methanolobus psychrophilus* R15 genome encompassing MetPsy-C1 and the remnants of a second casposons copy. The exact coordinates of the depicted regions are indicated in the figure. Genes are colored according to the scheme provided in Figure 2.

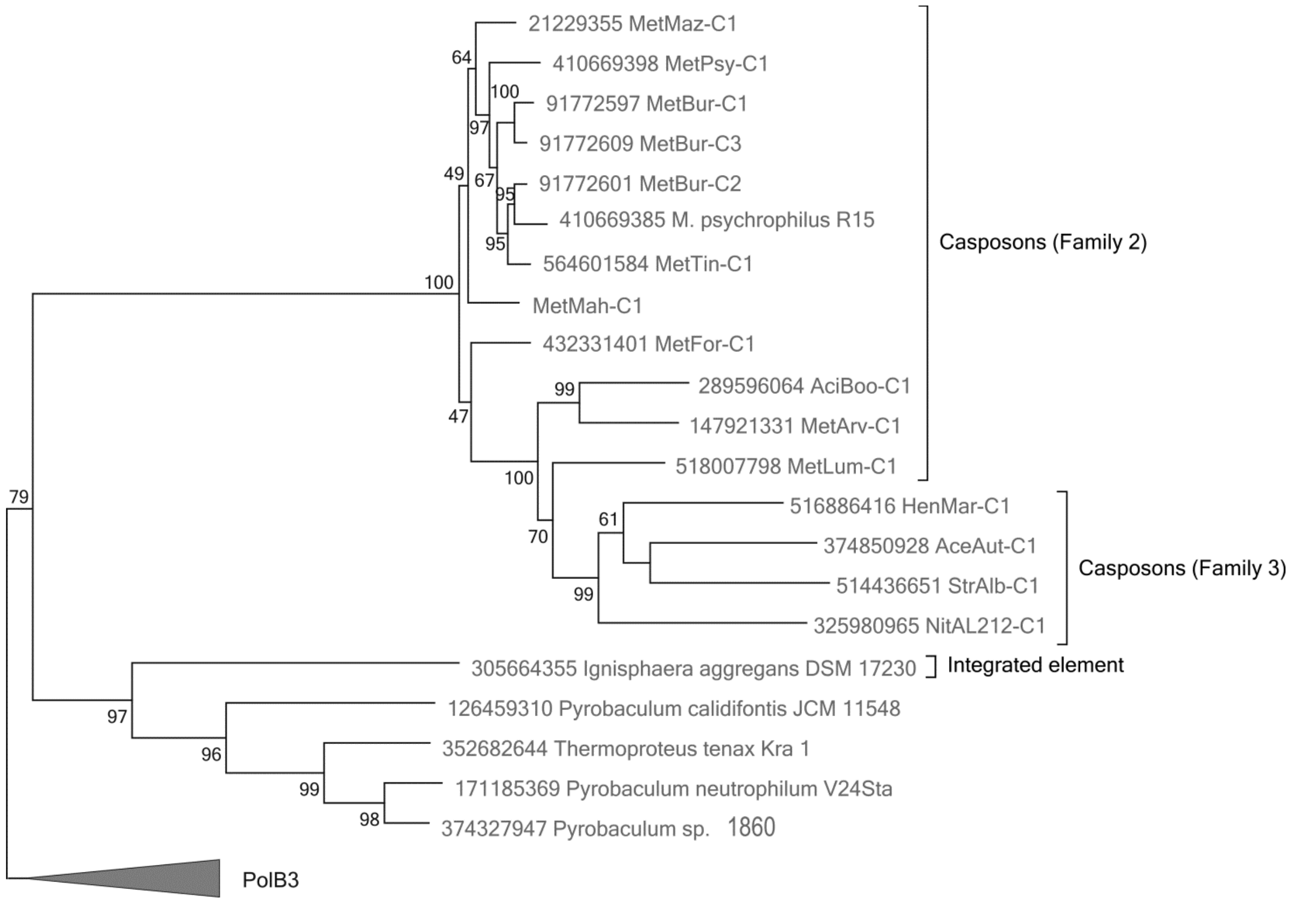

Figure S4. Phylogeny of RNA-primed type B DNA polymerases. The tree shows the relationship between the PolB proteins encoded by casposons of groups 2 and 3. Blasp analysis has shown that these casposon polymerases are most closely related to archaeal PolBs of group 3 (PolB3), which has been used as an outgroup in this tree. Numbers at the branch points represent RELL (resampling of estimated log-likelihoods)-like local support values calculated by FastTree.

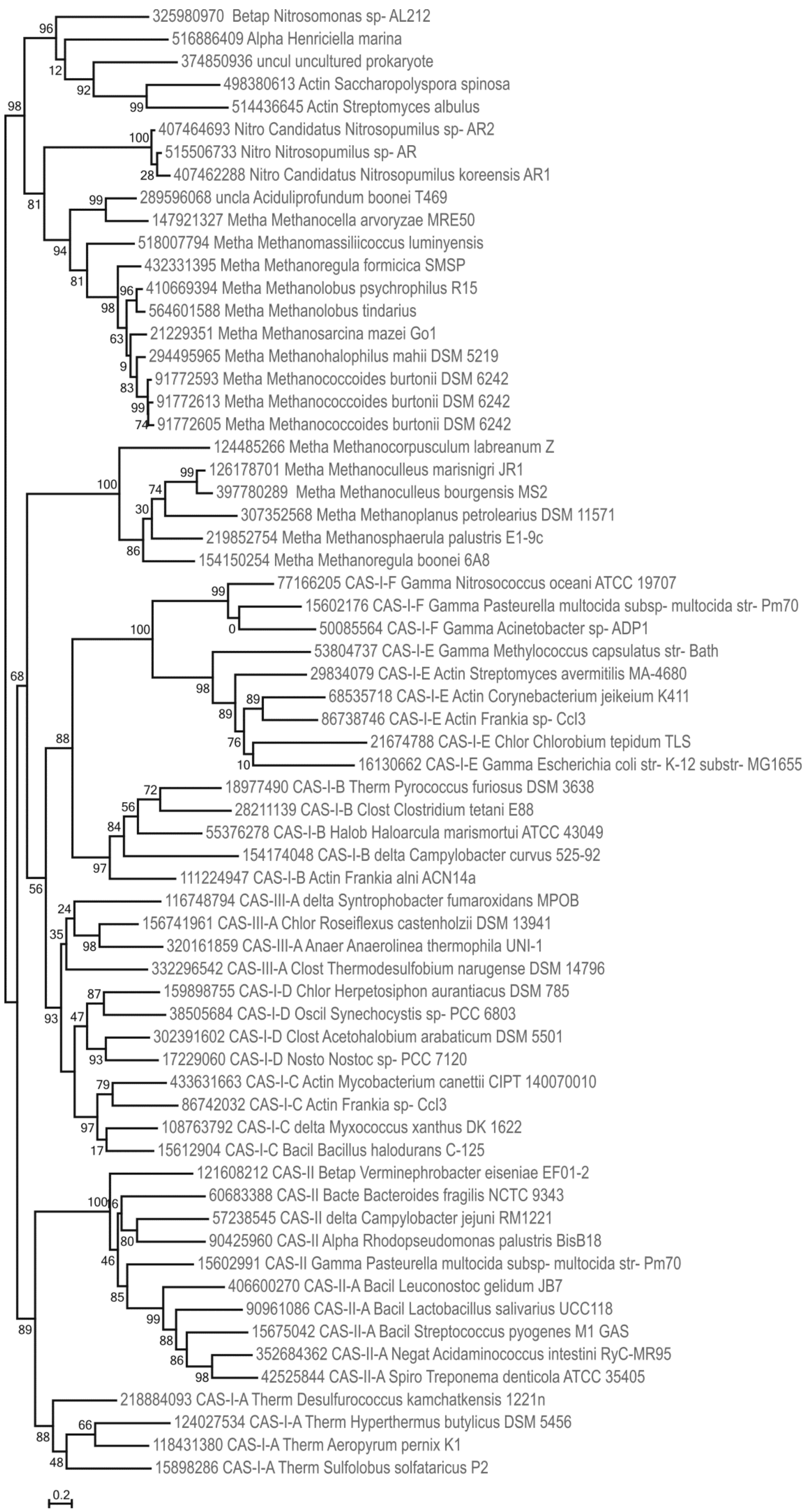

Figure S5. Phylogeny of Cas1 proteins. The tree is an expanded version of the tree shown in Figure 1. For each organism five letter code of taxonomy of the respective phylum (order) is indicated as well as the subtype of the CRISPR-Cas system. Numbers at the branch points represent RELL (resampling of estimated log-likelihoods)-like local support values calculated by FastTree.

SUPPLEMENTARY TABLES

Supplementary Table S1. Major characteristics of bacterial and archaeal casposons.

Name	Organism	Accession number	Coordinates	Size, bp	TIR	TSD	Overlap[a]	Target
**Archaea, Thaumarchaeota, Nitrosopumilales**								
NitAR-C1	Nitrosopumilus sp. AR	NZ_AJVJ01000035	1..12820	12,820	14*	-	-	-
NitSJ-C1	Nitrosopumilus sp. SJ	NZ_AJVI01000008	52214..60400	8,187	68	21	8/6	EF-2 (504775946)
NitAR1-C1	Candidatus Nitrosopumilus koreensis AR1	NC_018655	655308..663492	8,185	68	21	8/6	EF-2 (407462286)
NitAR2-C1	Candidatus Nitrosopumilus AR2	NC_018656	678437..686085	7,649	71	2	0	EF-2 (407464682)
**Archaea, Euryarchaeota, Thermoplasmatales, Aciduliprofundum**								
AciBoo-C1	Aciduliprofundum boonei T469	NC_013926	380320..389403	9,084	40	15	4/4	tRNA-Pro
**Archaea, Euryarchaeota, Methanomicrobia, unclassified**								
MetLum-C1	Methanomassiliicoccus luminyensis B10	NZ_CAJE01000015	159877..174942	15,066	29	17	4/0	intergenic
**Archaea, Euryarchaeota, Methanomicrobia, Methanomicrobiales**								
MetFor-C1	Methanoregula formicicum SMSP	NC_019943	1964119..1978982	14,864	91	14	0	tRNA-Leu
**Archaea, Euryarchaeota, Methanomicrobia, Methanocellales**								
MetArv-C1	Methanocella arvoryzae MRE50	NC_009464	2695218..2709760	14,543	134	14	0	tRNA-Arg
**Archaea, Euryarchaeota, Methanomicrobia, Methanosarcinales**								
MetPsy-C1	Methanolobus psychrophilus R15	NC_018876	190354..205199	14,846	56	-	-	intergenic
MetMah-C1	Methanohalophilus mahii DSM 5219	NC_014002	1316516..1332881	16,366	30	27	13/0	tRNA-Leu
MetMaz-C1	Methanosarcina mazei Go1	NC_003901	3946601..3956653	10,053	31	14	0	intergenic
MetTin-C1	Methanolobus tindarius DSM 2278	NZ_AZAJ01000001	3015418..3028607	ca. 13,190	25[#]	22	3/-	tRNA-Leu
MetBur-C1	Methanococcoides burtonii DSM 6242	NC_007955	566998..573184	6,187	-	-	-	intergenic
MetBur-C2	Methanococcoides burtonii DSM 6242	NC_007955	578143..584155	6,013	-	-	-	intergenic
MetBur-C3	Methanococcoides burtonii DSM 6242	NC_007955	586871..592908	6,038	-	-	-	intergenic
**Bacteria, Actinobacteria, Actinobacteridae, Actinomycetales**								
StrAlb-C1	Streptomyces albulus CCRC 11814	NZ_AROY02000134	18..16195	16,178	116	18	5/7	intergenic
**Bacteria, Proteobacteria, Alphaproteobacteria, Rhodobacterales**								
HenMar-C1	Henriciella marina DSM 19595	NZ_AQXT01000002	1654265..1670522	16,258	37	1	-	intergenic
**Bacteria, Proteobacteria, Betaproteobacteria, Nitrosomonadales**								
NitAL212-C1	Nitrosomonas sp. AL212	NC_015222	99718..114131	14,414	36	14	0	intergenic
**Environmental samples**								
AceAut-C1	Candidatus 'Acetothermum autotrophicum', fosmid JFF011 C09	AP011671	13064..33097	20,034	602	15	0/1	intergenic

* – incomplete; # – one of the TIRs is displaced to a different genomic locus; a – the number of nucleotides from the left and right TIRs which overlap the TSDs.

Supplementary Table S2. Annotation of the casposons.

NitAR-C1 from Nitrosopumilus sp. AR (NZ_AJVJ01000035)

Gene name	GI	Function	HHpred	Blast hit	Identity, E value
NAR_11980	515506719	Chitinase domain	Amicyanin coper-binding domain (3c75), P=99.7; Cell-wall hydrolase/chitinase domain (2kpn), P=98.6	Numerous bacterial and archaeal homologs	
NAR_11985	515506720	Bacterial Pleckstrin homology domain (bPH_3[pfam14470])	Pleckstrin homology domain (3hsa), P=99.8	Staphylococcus phage X2 (YP_240856);	54/196(28%), 2e-08
				Numerous bacterial homologs; Chryseobacterium sp. CF314 (WP_007843557)	62/185(34%), 2e-10
NAR_11990	515506721	SIR2-like domain (pfam13289)	SIR2-AF2, transcriptional regulatory protein (1ma3), P=95.4	Numerous bacterial homologues; Acetohalobium arabaticum DSM 5501 (YP_003827338)	73/234(31%), 2e-24
NAR_11995	515506722		HST2 histone deacetylase (1q1a); P=95.4 [N-terminal]		
NAR_12000	515506723				
NAR_12005	515506724	DNA_pol_B_2[pfam03175]	DNA polymerase delta catalytic subunit (3iay), phi29 (2py5), P=100	His1 virus (YP_529524) [also in His2, ABV, AP50, Wip1]	161/619(26%), 8e-31
NAR_12010	515506725			close homologues only in some Thaumarchaea	
NAR_12015	515506726			close homologues only in some Thaumarchaea	
NAR_12020	515506727			close homologues only in some Thaumarchaea	
NAR_12025	515506728			close homologues only in some Thaumarchaea	
NAR_12030	515506729			Methanolobus tindarius (WP_023846326)	54/223(24%), 9e-05
NAR_12035	515506730			close homologues only in some Thaumarchaea	
NAR_12040	515506731				
NAR_12045	515506732				
NAR_12050	515506733	Cas_Cas1 (pfam01867), E=5.1e-49	A.fulgidus Cas1 (4n06), P=100	Closest homologs in casposons	
NAR_12055	515506734				

NitAR2-C1 from Candidatus Nitrosopumilus AR2 (NC_018656)

Gene name	GI	Function	HHpred	Blast hit	Identity, E value
NSED_04085	407464683				
NSED_04090	407464684	DNA_pol_B_2[pfam03175], E=1.3e-19	phi29 (2py5), P=100; DNA polymerase delta catalytic subunit (3iay)	His2 virus (YP_529644) [His1, ABV, AP50, Wip1]	141/521(27%), 5e-32
NSED_04095	407464685			close homologues only in some Thaumarchaea	
NSED_04100	407464686			close homologues only in some Thaumarchaea	
NSED_04105	407464687	possibly HTH	some similarity to MazE antitoxin (1mvf), P=82.6	close homologues only in some Thaumarchaea	
NSED_04110	407464688			close homologues only in some Thaumarchaea	
NSED_04115	407464689	Zn-binding domain (C-terminal)	ZBD of DdRp (4ayb), P=98.8	close homologues only in some Thaumarchaea	
NSED_04120	407464690			homologues only in some Thaumarchaea, but distant homologs in bacteria; Nitrosoarchaeum limnia (WP_007402974)	29/121(24%), 1e-02
NSED_04125	407464691				
NSED_04130	407464692			Thaumarchaeota archaeon N4 (CDI04944); numerous homologues in bacteria annotated as methyltransferases	40/148(27%), 1e-08
NSED_04135	407464693	Cas_Cas1 (pfam01867), E=5.0e-45	T. maritima Cas1 (3lfx), P=100	Closest homologs in casposons	
NSED_04140	407464694			Some similarity to SSV7 VP3 (YP_003331516)	32/96(33%), 4e-02

NitSJ-C1 from Nitrosopumilus sp. SJ (NZ_AJVI01000008), identical to NitAR1-C1 from Candidatus Nitrosopumilus koreensis AR1 (NC_018655)

Gene name	GI	Function	HHpred	Blast hit	Identity, E value
NSJ_08960	504775947				
NSJ_08965	504775948	Cas_Cas1 (pfam01867)	T. maritima Cas1 (3lfx), P=100	Closest homologs in casposons	
NSJ_08970	504775949				

NSJ_08975	504775950	Zn-binding domain		close homologues only in some Thaumarchaea	
NSJ_08980	504775951				
NSJ_08985	504775952			homologues only in Cenarchaeum symbiosum A (YP_876344)	53/207(26%), 4e-06
NSJ_08990	504775953	Zn-binding domain (C-terminal)	ZBD of DdRp (4ayb), P=98.7	close homologues only in some Thaumarchaea	
NSJ_08995	504775954	wHTH protein	S. aureus MECI (1okr), P=96.5	close homologues only in some Thaumarchaea	
NSJ_09000	504775955		some similarity to MazE antitoxin (1mvf), P=81.7	close homologues only in some Thaumarchaea	
NSJ_09005	515506383			close homologues only in some Thaumarchaea	
NSJ_09010	515506384			close homologues only in some Thaumarchaea	
NSJ_09015	504775958	DNA_pol_B_2[pfam03175]	DNA polymerase delta catalytic subunit (3iay), phi29 (2py5), P=100	His1 virus (YP_529524) [His2, ABV, AP50, Wip1]	156/625(25%), 6e-31
NSJ_09020	504775959	RecG-related helicase	3lmm (uncharacterized), P=100; RecG from Nitrosomonas europaea (2kyy), P=99.9	numerous bacterial homologues; Mesorhizobium sp. STM 4661 (WP_006332744)	56/156(36%), 9e-10

AciBoo-C1 from Aciduliprofundum boonei T469 (NC_013926)

Gene name	GI	Function	HHpred	Blast hit	Identity, E value
Aboo_0387	289596064	PolB-HTH	DNA polymerase delta catalytic subunit (3iay), phi29, herpesvirus, etc., P=100; C-term HTH (3hot), P=95.2	Closest homologs in group 2 and 3 casposons	
Aboo_0388	289596065	HNH endonuclease (pfam01844)	HNH from phage P1 (3plw), P=97	Closest homologs in group 2 and 3 casposons	
Aboo_0389	289596066	HTH protein	HTH protein from Agrobacterium (1l3l), P=96.2	Closest homologs in group 2 and 3 casposons	
Aboo_0390	289596067	HTH_ArsR (COG1733)	wHTH from Pyrococcus horikoshii (1ub9), P=97)	remote similarity to bacterial proteins	
Aboo_0391	289596068	Cas1-HTH	A.fulgidus Cas1 (4n06), P=100; C-terminal HTH motif (4l5e), P=94.4	Closest homologs in casposons	
Aboo_0392	289596069	N6_Mtase (pfam02384)	type I RM Mtase (3lkd), P=100	Thaumarchaeota archaeon N4 (CDI05316)	262/954(27%), 8e-89

MetFor-C1 from Methanoregula formicicum SMSP (NC_019943)

Gene name	GI	Function	HHpred	Blast hit	Identity, E value
Metfor_2015	432331393	Coiled-coil (Cterm)		conserved in bacteria; Halanaerobium praevalens DSM 2228 (YP_005836384)	211/615(34%), 2e-78
Metfor_2016	432331394	HNH (C-term) - Cas9-like	C-terminal (469-596) domain related to HNH domain of Cas9 from S.pyogenes (4oo8), P=91.7	highly conserved in bacteria; Anabaena cylindrica PCC 7122 (YP_007155105)	145/612(24%), 2e-23
Metfor_2017	432331395	Cas1-HTH	A.fulgidus Cas1 (4n06), P=100; C-terminal HTH motif (1x3u), P=93.5	Closest homologs in casposons	
Metfor_2018	432331396	wHTH (Nterm), HEAT repeat (Cterm)	wHTH (2o03), P=89.1; HEAT repeat domain (1oyz), P=95.8	Closest homologs in group 2 casposons	
Metfor_2019	432331397	RHH	phage P22 Arc (1baz), P=97.2	Methanolobus psychrophilus R15 (YP_006921753)	23/44(52%), 7e-07
Metfor_2020	432331398	HTH protein	HTH protein from Agrobacterium (1l3l), P=96.5	Closest homologs in group 2 and 3 casposons	
Metfor_2021	432331399	wHTH	wHTH (2ia0), P=92	Closest homologs in group 2 and 3 casposons	
Metfor_2022	432331400	HNH	HNH of phage T4 EndoVII (1e7l), P=95.7	Closest homologs in group 2 and 3 casposons	
Metfor_2023	432331401	PolB-HTH	DNA polymerase delta catalytic subunit (3iay), phi29, herpesvirus, etc., P=100; C-term HTH (1x3u), P=92.1	Closest homologs in group 2 and 3 casposons	
Metfor_2024	432331402	HEAT repeats (pfam13646)	Importin-like HEAT repeat protein (3w3w), P=100		

MetArv-C1 from Methanocella arvoryzae MRE50 (NC_009464)

Gene name	GI	Function	HHpred	Blast hit	Identity, E value
RCIX16	147921324	ISNCY (flanked by inverted 15 nt repeats and 2 nt DR); DDE_Tnp_1[pfam01609]	Tn5 from E. Coli (1mus), P=99	Methanocella conradii HZ254 (YP_005380778)	161/227(71%), 1e-112
RCIX15	147921325	Sir2-like protein [pfam13289]	NAD-dependent deacetylase, SIR2 from T. maritima (3pdh), P=99.7	Bacillus thuringiensis (WP_000066156)	166/558(30%), 5e-65
RCIX13	147921326	N-terminal rubrerythrin-like domain	Rubrerythrin from D. vulgaris (1lko), P=94.2	Enterobacter cloacae UCI 24 (EUL87089)	45/181(25%), 3e-09
RCIX12	147921327	Cas1-HTH	A.fulgidus Cas1 (4n06), P=100; C-terminal HTH motif (1x3u), P=94.3	Closest homologs in casposons	
RCIX11	147921328	N-terminal wHTH domain (lineage-	wHTH protein from P.horikoshii (1ub9), P=95.1	Methanocella arvoryzae MRE50 (YP_685477)	84/328(26%), 3e-21

		specific)			
RCIX10	147921329	N-terminal HTH (LuxR-like)	HTH protein from S.aureus (2rnj), P=96.5	Closest homologs in group 2 and 3 casposons	
RCIX9	147921330	HNH, Zn-binding protein (C4)		Closest homologs in group 2 and 3 casposons	
RCIX8	147921331	PolB-HTH	DNA polymerase delta catalytic subunit (3iay), phi29, herpesvirus, etc., P=100; C-term HTH (4g6d), P=89.6	Closest homologs in group 2 and 3 casposons	
RCIX6	147921332				
RCIA1	147921333	N-terminal AN1-like Zinc finger (pfam01428)	Zn finger from A.thaliana (1wfh), P=99.5	Archaeoglobus veneficus SNP6 (YP_004342834)	48/202(24%), 2e-06
RCIX5	147921334				

MetPsy-C1 from Methanolobus psychrophilus R15 (NC_018876)

Gene name	GI	Function	HHpred	Blast hit	Identity, E value
Mpsy_0178	410669388	HNH[pfam01844]	HNH from Geobacter metallireducens (2qgp), P=99.2	Distant homologues in other archaea and bacteria; Halorubrum aidingense (WP_007998973)	34/84(40%), 4e-10
Mpsy_0179	410669389	N-terminal RHH domain	RHH from E.coli (2ay0), P=99.4	Archaeoglobus profundus DSM 5631 (YP_003400877)	19/62(31%), 7e-03
Mpsy_0180	410669390				
Mpsy_0181	410669391			Methanolobus tindarius (WP_023846527)	49/80(61%), 2e-15
Mpsy_0182	410669392				
Mpsy_0183	410669393				
Mpsy_0184	410669394	Cas1-HTH	A.fulgidus Cas1 (4n06), P=100; C-terminal HTH motif (1x3u), P=94.2	Closest homologs in casposons	
Mpsy_0185	410669395	wHTH (Nterm), HEAT repeat (Cterm)	wHTH (1r1u), P=94.5; HEAT repeat domain (1oyz), P=95.3	Closest homologs in group 2 casposons	
Mpsy_0186	410669396				
Mpsy_0187	410669397	HTH	HTH from Sinorhizobium meliloti (1x3u), P=96.6	Closest homologs in group 2 and 3 casposons	
ORF1	7348..7677	HNH[Cdd:pfam01844]	HNH from Geobacter metallireducens (2qgp), P=98.4	Closest homologs in group 2 and 3 casposons	
Mpsy_0188	410669398	PolB-HTH	DNA polymerase delta catalytic subunit (3iay), phi29, herpesvirus, etc., P=100; C-term HTH (4l5e), P=87.9	Closest homologs in group 2 and 3 casposons	
Mpsy_0189	410669399	AP endonuclease	AP endonuclease from P.aeruginosa (4jg3), P=100	Numerous bacterial and archaeal homologs; Methanosarcina barkeri str. Fusaro (YP_305346)	112/220(51%), 2e-69
Mpsy_0190	410669400	GIY-YIG nuclease	Endonuclease II; GIY-YIG from phage T4 (2wsh), P=97.3	Numerous bacterial homologs; Natranaerobius thermophilus JW/NM-WN-LF (YP_001916976)	129/279(46%), 2e-83
Mpsy_0191	410669401	Uracil DNA glycosylase [pfam03167]	UDG from Thermus thermophilus (2d3y), P=100	Bacterial homologues; Methylobacterium sp. 4-46 (YP_001768434)	41/197(21%), 2e-05
Mpsy_0192	410669402	PolB fragment (C-terminal HTH)			
Mpsy_0193	410669403	AP endonuclease	AP endonuclease from P.aeruginosa (4jg3), P=100	Numerous bacterial and archaeal homologs; Methanosarcina barkeri str. Fusaro (YP_305346)	112/220(51%), 2e-69
Mpsy_0194	410669404	GIY-YIG nuclease	Endonuclease II; GIY-YIG from phage T4 (2wsh), P=97.3	Numerous bacterial homologs; Natranaerobius thermophilus JW/NM-WN-LF (YP_001916976)	129/279(46%), 2e-83

MetMaz-C1 from Methanosarcina mazei Go1 (NC_003901)

Gene name	GI	Function	HHpred	Blast hit	Identity, E value
MM_3247	21229349	Pentapeptide repeat protein [pfam00805]	Pentapeptide repeat protein from Cyanothece (2o6w), P=99.8	Numerous bacterial homologs; Fischerella muscicola (WP_016862898)	114/297(38%), 6e-46
MM_3248	21229350	DUF4145[pfam13643]		Acinetobacter baumannii 318814 (EXH91314)	57/184(31%), 6e-06
MM_3249	21229351	Cas1-HTH	A.fulgidus Cas1 (4n06), P=100; C-terminal HTH motif (1x3u), P=93.7	Closest homologs in casposons	
MM_3250	21229352	wHTH (Nterm), HEAT repeat (Cterm)	wHTH (2lnb), P=93.2; HEAT repeat domain (1oyz), P=94.3	Closest homologs in group 2 casposons	
MM_3251	21229353	HTH	HTH from Sinorhizobium meliloti (1x3u), P=96.7	Closest homologs in group 2 and 3 casposons	
MM_3252	21229354	HNH[pfam01844]	HNH from Geobacter metallireducens (2qgp), P=98.3	Closest homologs in group 2 and 3 casposons	
MM_3253	21229355	PolB-HTH	DNA polymerase delta catalytic subunit (3iay), phi29, herpesvirus, etc., P=100; C-term HTH (1tty), P=82.2	Closest homologs in group 2 and 3 casposons	
MM_3254	21229356	HEAT protein		Bacterial homologs; Aminicenantes bacterium SCGC AAA252-O11 (WP_020262809)	73/226(32%), 2e-25

MetLum-C1 from Methanomassiliicoccus luminyensis B10 (NZ_CAJE01000015)

Gene name	GI	Function	HHpred	Blast hit	Identity, E value
WYS_09850	518007794	Cas1-HTH	A.fulgidus Cas1 (4n06), P=100; C-terminal HTH motif (1x3u), P=94.3	Closest homologs in casposons	
WYS_09855	518007795				
WYS_09860*	518007796	HTH	HTH from Sinorhizobium meliloti (1x3u), P=95.6	Closest homologs in group 2 and 3 casposons	
WYS_09865	518007797	HNH	HNH from phage P1 (3plw), P=98.1	Closest homologs in group 2 and 3 casposons	
WYS_09870	518007798	PolB-HTH	DNA polymerase delta catalytic subunit (3iay), phi29, herpesvirus, etc., P=100; C-term HTH (1x3u), P=90.7	Closest homologs in group 2 and 3 casposons	
WYS_09875	518007799				
WYS_09880*	518007800	ZBD (2XC4)	Many hits to ZF proteins		
ORF1	7387..7608				
ORF2	7727..8593	Fibronectin type 3 domain (COG3401)	Neogenin; cell adhesion, Mus musculus (4bq6), P=99.9	Aciduliprofundum sp. MAR08-339 (YP_007246776)	89/201(44%), 2e-29
WYS_09890	518007802				
WYS_09895	518007803	DNA-binding swapped-hairpin barrel protein	Swapped-hairpin barrel protein from B.subtilis (1yfb), P=99.7	Nitrosoarchaeum limnia (WP_010190738)	15/35(43%), 6e-02
WYS_09900	518007804	AP endonuclease	AP endonuclease from P.aeruginosa (4jg3), P=100	Numerous bacterial and archaeal homologs; Methanosarcina acetivorans C2A (NP_618490)	89/227(39%), 6e-54
WYS_09905	518007805			Turneriella parva DSM 21527 (YP_006441700)	45/111(41%), 3e-18
ORF3	11228..12595	Restriction endonuclease	Restriction endonuclease from Mycobacterium (4f0q), P=95.6	Multiple bacterial homologs; Niastella koreensis GR20-10 (YP_005009107)	261/456(57%), 0
WYS_09915	518007807			Multiple bacterial homologs; Niastella koreensis GR20-10 (YP_005009106)	54/123(44%), 1e-30
WYS_09920	518007808			Multiple bacterial homologs; Niastella koreensis GR20-10 (YP_005009105)	48/94(51%), 3e-22
ORF4	13318..13572				
WYS_09925	518007809			Multiple bacterial homologs; Moorella thermoacetica ATCC 39073 (YP_430511)	139/415(33%), 2e-54

MetMah-C1 from Methanohalophilus mahii DSM 5219 (NC_014002)

Gene name	GI	Function	HHpred	Blast hit	Identity, E value
Mmah_1308	294495961	Sir2-like protein [pfam13289]	NAD-dependent deacetylase, SIR2 from T. maritima (1yc5), P=99.9	Clostridium] glycolicum (WP_018590993)	119/368(32%), 2e-52
Mmah_1309	294495962			Methanolobus tindarius (WP_023844956)	64/138(46%), 3e-38
Mmah_1310	294495963	Immunoglobulin A1 protease	Putative immunoglobulin A1 protease (3n6z), P=100; PKD domain (4l9d), P=99.6; Cellulosomal-scaffolding protein A (3kcp), P=95.1	Methanosalsum zhilinae DSM 4017 (YP_004616399)	225/527(43%), 2e-84
Mmah_1311	294495964	Cellulosome anchoring protein	Cellulosome anchoring protein from Clostridium thermocellum (3kcp_B), P=97.2	Methanohalobium evestigatum Z-7303 (YP_003727885)	95/224(42%), 4e-46
Mmah_1312	294495965	Cas1-HTH	A.fulgidus Cas1 (4n06), P=100; C-terminal HTH motif (1x3u), P=93.9	Closest homologs in casposons	
Mmah_1314	294495966	wHTH (Nterm), HEAT repeat (Cterm)	wHTH (3tgn), P=95.9; HEAT repeat domain (3ltj), P=84.2	Closest homologs in group 2 casposons	
Mmah_1315	294495967	HTH	HTH (1l3l), P=96.9	Closest homologs in group 2 and 3 casposons	
Mmah_1316	294495968	HNH	HNH from Geobacter metallireducens (2qgp), P=98.4	Closest homologs in group 2 and 3 casposons	
ORF1	9525..12227	PolB (internal stop codon)		Closest homologs in group 2 and 3 casposons	
Mmah_1318	294495969	coiled-coil			
Mmah_1319	294495970	KAP P-loop ATPase [pfam07693]	CD hit: KAP_NTPase[pfam07693], E=1.1e-34	Numerous bacterial homologs; Leptospira santarosai (WP_004473541)	282/756(37%), 7e-157

MetTin-C1 from Methanolobus tindarius DSM 2278 (NZ_AZAJ01000001)

Gene name	GI	Function	HHpred	Blast hit	Identity, E value
MettiDRAFT_2892	564601581			Clostridium difficile (WP_009896147)	48/188(26%), 4e-08

MettiDRAFT_2893	564601582	Cas4-like nuclease	Hit to Cas4 protein of S. Solfataricus (4ic1), P=88	Ktedonobacter racemifer (YP_004004503)	90/295(31%), 1e-27
MettiDRAFT_2894	564601583			MetPsy-T1 (410669391)	48/80(60%), 3e-16
MettiDRAFT_2895	564601584	PolB-HTH	DNA polymerase delta catalytic subunit (3iay), phi29, herpesvirus, etc., P=100; C-term HTH (2o8x), P=94.5	Closest homologs in group 2 and 3 casposons	
MettiDRAFT_2896	564601585	HNH_2[pfam13391]	HNH from Geobacter metallireducens (2qgp), P=98.6	Closest homologs in group 2 and 3 casposons	
MettiDRAFT_2897	564601586	HTH	HTH from Sinorhizobium meliloti (1x3u), P=96.7	Closest homologs in group 2 and 3 casposons	
MettiDRAFT_2898	564601587	wHTH (Nterm), HEAT repeat (Cterm)	wHTH (3tgn), P=95.6; HEAT repeat domain (3ltj), P=93.5	Closest homologs in group 2 casposons	
MettiDRAFT_2899	564601588	Cas1-HTH	A.fulgidus Cas1 (4n06), P=100; C-terminal HTH motif (1x3u), P=93.7	Closest homologs in casposons	

MetBur-C1 from Methanococcoides burtonii DSM 6242 (NC_007955)

Gene name	GI	Function	HHpred	Blast hit	Identity, E value
Mbur_0560	91772593	Cas1-HTH	A.fulgidus Cas1 (4n06), P=100; C-terminal HTH motif (1x3u), P=94.2	Closest homologs in casposons	
Mbur_0561	91772594	wHTH (Nterm), HEAT repeat (Cterm)	wHTH (4hbl), P=94.2; HEAT repeat domain (1oyz), P=95	Closest homologs in group 2 casposons	
Mbur_0562	91772595	HTH	HTH from Sinorhizobium meliloti (1x3u), P=96.7	Closest homologs in group 2 and 3 casposons	
Mbur_0563	91772596	HNH[pfam01844]	HNH from Geobacter metallireducens (2qgp), P=98.5	Closest homologs in group 2 and 3 casposons	
Mbur_0564	91772597	PolB-HTH	DNA polymerase delta catalytic subunit (3iay), phi29, herpesvirus, etc., P=100; C-term HTH (PF13518), P=88	Closest homologs in group 2 and 3 casposons	

AceAut-C1 from Candidatus 'Acetothermum autotrophicum', fosmid JFF011 C09 (AP011671)

Gene name	GI	Function	HHpred	Blast hit	Identity, E value
HGMM_F11C09C11	374850928	PolB-HTH	DNA polymerase delta catalytic subunit (3iay), phi29, herpesvirus, etc., P=100; C-term: Phage 434 Cro-like HTH (1zug), P=93	Closest homologs in group 2 and 3 casposons	
ORF1	complement(3974..4399)	HNH		Methanoregula formicica SMSP (YP_007249543)	25/83(30%), 1.3
HGMM_F11C09C12	374850929	HTH protein	HTH protein from Agrobacterium (1l3l), P=96.5	homologs in group 2 and 3 casposons	
HGMM_F11C09C13	374850930	Nucleoside 2-deoxyribosyltransferase	Many hits; 1t1j, P=97.8		
HGMM_F11C09C14	374850931	SM-like protein [pfam01423]	SM-like protein from Methanothermobacterthermautotrophicus (1mgq), P=97.2		
HGMM_F11C09C15	374850932	ParB	ParB from Thermus thermophilus (1vz0), P=100	Micromonospora sp. ATCC 39149 (WP_007076246)	42/124(34%), 7e-09
HGMM_F11C09C16	374850933				
HGMM_F11C09C17	374850934				
HGMM_F11C09C18	374850935			Sulfobacillus thermosulfidooxidans (WP_020375118)	58/198(29%), 9e-19
HGMM_F11C09C19	374850936	Cas1-Zf	A.fulgidus Cas1 (4n06), P=100; C-terminal Zfmotif (1lv3), P=86	Closest homologs in casposons	
HGMM_F11C09C20	374850937	Acetyltransferase[pfam00583]/tRNA-guanine transglycosylase	N: GCN5-N-acetyltransferase (2cnt), P=99.8; C: Queuine tRNA-ribosyltransferase (4e2v), P=100	Oscillatoria acuminata PCC 6304 (YP_007087902)	213/457(47%), 9e-143
HGMM_F11C09C21	374850938	Dehydroquinase class II (pfam01220)	3-dehydroquinase from C. Albicans (3kip), P=90.6	Oscillatoria acuminata PCC 6304 (YP_007087901)	88/223(39%), 6e-49
HGMM_F11C09C22	374850939	ATPase (helicase loader)	Helicase loader from Aquifex aeolicus (3ec2), P=99.8	Methanocaldococcus sp. FS406-22 (YP_003457567)	51/175(29%), 1e-10
HGMM_F11C09C23	374850940	Cdc6-like AAA+ ATPase, C-terminal coiled-coil domain	N (23-416): Cdc6-like origin recognition complex (1w5s), P=95.7; C-terminal coiled-coil domain	Oscillatoria acuminata PCC 6304 (YP_007087900)	444/1361(33%), 0
HGMM_F11C09C24	374850941	Ectonucleotide pyrophosphatase	Ectonucleotide pyrophosphatase from Sinorhizobium meliloti (3szy), P=99.7	Methanocaldococcus sp. FS406-22 (YP_003457569)	87/283(31%), 1e-33
HGMM_F11C09C25	374850942	HTH protein [pfam14338]	HTH protein from B. subtilis (1on2), P=96.8	Thermosipho africanus (WP_004103278)	69/224(31%), 4e-25
HGMM_F11C09C26	374850943	FtsK/HerA-like helicase (COG0433)	VirB4 ATPase from Thermoanaerobacter pseudethanolicus (4ag6), P=100	Methanocaldococcus sp. FS406-22 (YP_003457570)	194/390(50%), 1e-130

StrAlb-C1 from Streptomyces albulus CCRC 11814 (NZ_AROY02000134)

Gene name	GI	Function	HHpred	Blast hit	Identity, E value
K530_27579	514436645	Cas1	A.fulgidus Cas1 (4n06), P=100	Closest homologs in casposons	
K530_53465	529138494				

ORF1	complement(1729..1998)				
ORF2	complement(1995..2309)				
K530_27584	514436646				
K530_27589	514436647			Bacillus cytotoxicus NVH 391-98 (YP_001373573)	33/89(37%), 1e-03
ORF3	complement(3573..3869)				
ORF4	4272..5153	AAA ATPase [smart00382]	ABC ATPase from Geobacillus stearothermophilus (2olj), P=100	Archaeoglobus veneficus SNP6 (YP_004341389)	44/167(26%), 3e-05
K530_53470	529138496				
K530_27594*	514436648			Streptomyces collinus Tu 365 (YP_008390667)	87/321(27%), 1e-13
ORF5	6629..6925				
K530_53480	529138499				
ORF6	7117..7761				
K530_53485	529138500				
K530_27599	514436649				
K530_27604	514436650				
ORF7	9513..9920				
ORF8	10003..10278	HNH	Partial hit: HNH from Geobacter metallireducens gs-15 (2qgp), P=97.4		
K530_27609	514436651	PolB	DNA polymerase delta catalytic subunit (3iay), phi29, herpesvirus, etc., P=100	Closest homologs in group 2 and 3 casposons	
K530_53490	529138503	ZBD (C4)	ZBD (4mb7), P=94.3		
ORF9	complement(13627..13971)	ZBD	Ada-like ZBD (1u8b), P=96.2	Prevotella sp. CAG:5226 (WP_02210236)	21/42(50%), 7e-04
K530_27614	514436652				
K530_53495*	529138504				
K530_53500	529138507			Conserved in Streptomyces; Streptomyces sp. MspMP-M5 (WP_018540923)	77/112(69%), 1e-33
K530_01207*	514430605	HTH_XRE[cd00093]	HTH from Bacillus caldolyticus (2b5a), P=99.5	Streptomyces albulus PD-1 (EXU89943)	277/277(100%)
K530_53505*	530788168	putative transcriptional regulator		Streptomyces albulus PD-1 (EXU89944)	69/69(100%)

NitAL212-C1 from Nitrosomonas sp. AL212 (NC_015222)

Gene name	GI	Function	HHpred	Blast hit	Identity, E value
NAL212_0248	325980964	SFI helicase (COG1112)	N: UPF1 helicase (2wjy), P=100; C: homing endonuclease from Bacillus phage 0305phi8-36 (3r3p), P=99.2	Rhodopseudomonas palustris BisB5 (YP_567730)	604/1550(39%), o
NAL212_0249	325980965	PolB-HTH	DNA polymerase delta catalytic subunit (3iay), phi29, herpesvirus, etc., P=100; C-term: transcriptional regulator VanUg (3t76), P=92.2	Closest homologs in group 2 and 3 casposons	
NAL212_0250	325980966	IS1595 (flanked by inverted 15 nt repeats); DDE_Tnp_IS1595[pfam12762]	RVE integrase of Rous sarcoma virus (4fw2), P=97.2	Multiple copies in Nitrosomonas sp. AL212; Acidithiobacillus caldus SM-1 (YP_004747527)	193/294(66%), 9e-128
NAL212_0251	325980967	HNH	Partial hit: HNH from Geobacter metallireducens gs-15 (2qgp), P=97.5	homologs in group 2 and 3 casposons	
NAL212_0252	325980968	HTH		Methanohalophilus mahii DSM 5219 (YP_003542460)	36/151(24%), 4e-03
NAL212_0253	325980969	HTH	TipA-like HTH from Streptomyces lividans (2vz4), P=96.7		
NAL212_0254	325980970	Zf-Cas1	N: C4 Zn finger from E.coli (1lv3), P=98.7; C: Thermotoga maritima Cas1 (3lfx), P=100	Closest homologs in casposons	
NAL212_0255	325980971			Vibrio cyclitrophicus (WP_016800302)	64/208(31%), 3e-10

HenMar-C1 from Henriciella marina DSM 19595 (NZ_AQXT01000002)

Gene name	GI	Function	HHpred	Blast hit	Identity, E value

F550_07975	516886403	Cas4-SFI helicase (COG1112)	N: Cas4-like from Sulfolobus solfataricus (4ic1), P=97.6; C: UPF1 helicase (2wjy), P=100	Leptolyngbya boryana (WP_017289541)	544/1403(39%), 0
ORF1	complement(4212..5417)	C-5 DNA methylase [Cdd:pfam00145]	DNA Mtase from Haemophilus aegyptius (3ubt), P=100	Syntrophobacter fumaroxidans MPOB (YP_845448)	186/394(47%), 1e-108
F550_07990	516886409	Cas1	Thermotoga maritima Cas1 (3lfx), P=100	Closest homologs in casposons	
F550_07995	516886411	HTH_MerR-like_sg6[cd04781]	MerR-like HTH (3hh0), P=94.4		
F550_08000	516886413	HTH	HTH (1l3l), P=96.3	Methanohalophilus mahii DSM 5219 (YP_003542460)	40/151(26%), 2e-02
F550_08005	516886414	HNH, ZBD (C4)		Methanohalophilus mahii DSM 5219 (YP_003542461)	17/49(35%), 0.37
F550_08010	516886416	PolB	DNA polymerase delta catalytic subunit (3iay), phi29, herpesvirus, etc., P=100	Closest homologs in group 2 and 3 casposons	
F550_08015	516886418				
F550_08020*	516886421	Ser/Thr kinase [cd04791]	Ser/Thr-protein kinase 6 (2j4z), P=100	Stigmatella aurantiaca DW4/3-1 (YP_003956746)	288/883(33%), 1e-128
ORF2	complement(14946..16124)	Cysteine dioxygenase type I	Cysteine dioxygenase type I (2gm6), P=98.3	Corallococcus coralloides DSM 2259 (YP_005369259)	78/265(29%), 5e-23
